# Single-Step Synthesis and Characterization of Non-Linear Tough and Strong Segmented Polyurethane Elastomer Consisting of Very Short Hard and Soft Segments and Hierarchical Side-Reacted Networks and Single-Step Synthesis of Hierarchical Hyper-Branched Polyurethane

**DOI:** 10.3390/molecules29071420

**Published:** 2024-03-22

**Authors:** Theodor Stern

**Affiliations:** Department of Chemical Engineering, Biotechnology and Materials, Faculty of Engineering, Ariel University, Ariel 40700, Israel; theodorst@ariel.ac.il

**Keywords:** polyurethane, structure–property relations, FTIR, NMR, hyper-branched polymer, polymerization mechanism, side reactions, network

## Abstract

Polyurethane elastomers are among the most versatile classes of industrial polymers—typically achieved through a two-step synthesis of segmented block copolymers, comprising very long and soft segments that provide elasticity and significantly long and hard segments that provide strength. The present research focused on the design of a single-step synthesis of a new segmented polyurethane consisting of very short soft and hard segments, crosslinked by preferentially side-reacted hierarchical tertiary oligo-uret network structures, thus exhibiting significant strength, elasticity, and toughness. Despite the theoretically linear structure, both FTIR and solid-state ^13^C NMR spectroscopy analyses indicated the quasi-equal presence of urethane groups and tertiary oligo-uret structures in the resulting polymer, indicating a preferential consecutive side reaction mechanism. Thermal analysis indicated the significant crystallization of soft segments consisting of only four ethylene oxide units, which was, hereby, demonstrated to occur via an extended chain mechanism. Tensile mechanical properties included significant strength, elasticity, and toughness. Increasing the soft segment length led to a decreased tertiary oligo-uret secondary crosslinking efficacy. The preferential hierarchical side reaction mechanism was, hereby, further confirmed through the synthesis of a completely new type of hyper-branched polymer via diisocyanate and a mono-hydroxy-terminated reagent. The structure–property relations and reaction mechanisms demonstrated in the present research can facilitate the design of new polyurethanes of enhanced performance and processing efficacy for a variety of novel applications.

## 1. Introduction

Polyurethanes are among the most important and highly versatile classes of industrial polymers, exhibiting a vast variety of applications ranging from very common consumer products, such as shoe soles, foams, textile fibers, coatings, and parts for the automotive industry, to biomedical applications, such as medical devices and artificial implants.

Polyurethanes are typically synthesized through the reaction between di-functional isocyanates and di-functional primary alcohols, leading to the formation of urethane-containing repeating polymer units of thermoplastic polyurethanes [1]. The use of aliphatic or aromatic diisocyanates in combination with very short diols results in the formation of highly rigid and strong polymers, which are highly suitable for applications that require such properties [1,2,3]. Nevertheless, all applications of polyurethanes require elasticity to some extent, with some demanding large deformations. Elastic polymers are commonly termed elastomers. Polyurethane elastomers are typically synthesized so as to consist of alternating hard and soft segments, preferably exhibiting microphase segregation due to purposefully designed significant differences in polarity [1,2,3].

The most commonly used soft segments are diol-terminated soft polymers of molecular weights in the range of thousands of g/mol (e.g., 2000–6000 g/mol), mostly diol-terminated polyethers or polyesters. The synthesis of a segmented polyurethane typically requires a two-step process, consisting of the preliminary synthesis of a macro-diisocyanate prepolymer (through the reaction of a diisocyanate with each of the two terminal hydroxyls), which is subsequently further reacted with additional di-functional reagents such as diols, diamines, or dicarboxylic acids, commonly termed as chain extenders, resulting in additional urethane, urea, or amide groups, respectively [1,2,3,4,5,6,7,8,9,10,11,12,13,14]. As the urethane groups, urea groups, and amide groups are relatively planar and rigid and have the ability of hydrogen bonding, they constitute the polymer’s hard segments and are the main source of the polymer’s strength. The less ordered soft segments stretch and align in the direction of an applied tensile stress and relax when the stress is released, thus creating the elastic properties of the polymer [1,2,3,4,5,6,7,8,9,10,11,12,13,14,15,16,17,18,19]. Although the reaction between a diol-terminated soft segment and a diisocyanate is sufficient for the formation of a polyurethane polymer [1,2], the above-described macro-diisocyanate and chain extension synthesis pathway is usually performed with the purpose of obtaining a longer hard segment, thus obtaining significantly strong elastomers, especially when using relatively long and soft segments [3,4,5,6,7,8,9,10,11,12,13,14,15,16,17,18,19]. A well-known example of this is Lycra^®^, which is a poly(ether urethane urea) segmented elastomer mostly applied as a textile fiber and also in some biomedical uses [20,21,22,23,24,25].

In polyurethane synthesis, the only theoretical chemical change occurring is the formation of the urethane groups and either urea or amide when chain-extended with a diamine or a dicarboxylic acid, respectively. Each of these groups contains one carbonyl, the presence and spectral location of which is highly analytically diagnostic for the monitoring and confirmation of the polymerization reaction occurrence and for the identification of the types of groups which are formed. FTIR spectroscopy analysis is among the most widely used analytical methods for the assessment of the polyurethane synthesis process and of the final product chemical structure, both in research and in industrial processes.

Nevertheless, most industrial polyurethanes contain significantly long and soft segments, thus resulting in a very high intensity of the analytical signals stemming from the very abundant ether or ester groups of said soft segments and, thus, a very low intensity of the analytical signals related to the carbonyls of the hard segment structures, due to their much lower concentration in the polymer. As a consequence, the accurate diagnostic identification of the different carbonyl types occurring in polyurethane hard segments is a challenging task in these materials. The ability to perform accurate diagnostic carbonyl type identification is also highly important for the correct assessment and analysis of side reactions that may occur in polyurethane synthesis, which may highly affect the final polymer properties and possible applications.

Polyurethane synthesis is accompanied by side reactions occurring predominantly between isocyanate groups and the secondary nitrogen groups (i.e., NH groups) of already formed urethane groups, due to the inherently very high reactivity of the isocyanate groups. This side reaction is long-known to result in the formation of allophanate structures [1,2,3]. 

Despite the very long-known tendency of diisocyanates to side-react with the NH groups of urethanes and ureas to form allophanates and biurets, respectively [1,2,3], very numerous polyurethane synthesis-related research studies over many decades have attributed the occurrence of two, and even multiple, distinct carbonyl-stretching absorbances in the FTIR spectra of polyurethanes to a combined presence and absence of hydrogen bonding between some portions of the urethane groups—without even considering the very possible occurrence of side reactions and, consequently, the possible formation of chemical structures containing additional carbonyl types, thus exhibiting different adjacent carbonyl-stretching absorbance locations. In a very early book entitled *Polyurethanes*—*Chemistry*, *Technology* and *Properties*, published in 1964 [26], the authors state as follows: “There is no doubt that isocyanates react with urethanes, but surprisingly few accounts occur in the literature of the isolation and characterization of a product. If the isocyanate reacts ‘normally’ with a urethane, the product will be a substituted allophanic ester” [26] (p. 108). It is even more surprising that, to the present day, most of the reported polyurethane synthesis-related research works using diisocyanates do not even mention the very high possibility of side reactions occurring—and mostly attribute the occurrence of two, or even multiple, distinct adjacent carbonyl-stretching FTIR absorbances to the effects of hydrogen bonding and the non-bonding of the urethane carbonyls [27,28,29,30,31,32,33,34].

It was recently demonstrated, though, that once allophanate structures are formed, they preferentially further consecutively side-react with additional isocyanate groups to form crosslinking hierarchical tertiary oligo-uret network structures [35,36]. The preferential formation of tertiary oligo-uret structures was further demonstrated to also occur in diisocyanate-derived polyurea synthesis [37,38] and was additionally found to be highly dependent on the polyurea synthesis pathway [38], the relatively mild and slow water–diisocyanate pathway predominantly and preferentially produces tertiary oligo-uret structures [38], while the extremely quick and highly exothermal diamine–diisocyanate synthesis pathway of the exact same polymer almost exclusively leads to the formation of biuret structures [37]. These tertiary oligo-uret structures, as also described in the referenced research works [35,36,37,38], may schematically be represented by the following: -N(R)C=ON(R)C=ON(R)C=ON(R)C=ON(R)… and so on (with R representing the diisocyanate R group, such as, for example, hexamethylene). The length of the tertiary oligo-uret structure depends on the number of hierarchical consecutive side reactions that occurred. All the nitrogens in the structure, except the last one, are tertiary nitrogens. 

Thus, the so-called linear polyurethanes are actually non-linear, due to the concomitant occurrence of these side reactions [1,2,3,26,35,36,37,38]. 

Side reactions are, by definition, commonly regarded as unwanted—and are, thus, named as side reactions, occurring beside the wanted reaction—and when occurring in significant proportion during polymer synthesis, they may significantly affect the final polymer properties and possible applications. This effect on the final polymer properties is especially accentuated in polyurethanes where side reactions may lead to significant polymer crosslinking [1,2,3,26,35,36,37,38]. 

As the above-described side reactions concomitantly occur during polyurethane synthesis, it would be highly beneficial to advantageously use these side reactions in the design and synthesis of new types of polyurethanes, with possibly new structures, properties, and applications. 

The presence of these highly complex hierarchical crosslinking networks—concomitantly inducing both strength and flexibility—may offer the possibility of significantly reducing the hard segment length while still obtaining the desired mechanical properties that are commonly obtained with very large, hard segments, which commonly also contain aromatic R groups. Since long, hard segments are typically obtained via a two-step prepolymer and chain extension process, designing a single-step polyurethane synthesis also has the distinct advantage of requiring the use of much reduced amounts of diisocyanate, which would be highly beneficial in terms of both safety and environmental considerations. Also, the compensating presence of the hierarchical tertiary oligo-uret crosslinking networks may offer the possibility of using aliphatic diisocyanates—instead of aromatic—which is also highly beneficial in the same context.

The present research focused on the design of a single-step synthesis and characterization of a new segmented polyurethane consisting of very short hard and soft segments, combining high flexibility and enhanced mechanical properties. The concept of the present research stems from the initial hypothesis that the above-described expected preferential formation of crosslinking tertiary oligo-uret network structures may increase the efficacy of a very short hard segment. Concomitantly, this formation may also enable the generation of significant elastic properties using a very short soft segment, due to a certain degree of flexibility in the long hierarchical tertiary oligo-uret crosslinking network structures. 

## 2. Results and Discussion

**Scheme 1:**
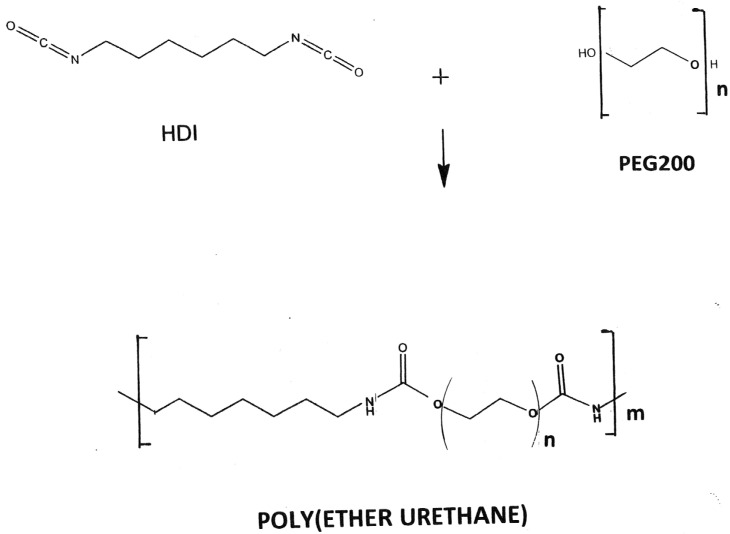
Schematic representation of the synthesis process and theoretical linear chemical structure of the poly(ether urethane), synthesized with hexamethylene diisocyanate (HDI) and PEG200 at a molar ratio of 1:1.

The synthesis of segmented polyurethane designed in the present research was performed using hexamethylene diisocyanate (HDI) and PEG200 at a molar ratio of 1:1. The theoretical linear structure of the resulting polymer is presented in Scheme 1. As observed in Scheme 1, the very short, hard segment consists of only two urethane groups in the polymer repeating unit, separated by the hexamethylene group of the original HDI. The soft segment of the polymer, i.e., PEG200, is very short when compared to industrial polyurethanes such as the well-known Lycra^®^, in which the soft segment consists of polytetramethylene glycol (PTMG) 2000 and the hard segment consists of two urethane groups and two urea groups in the polymer repeating unit [20,21,22,23,24,25].

**Figure 1:**
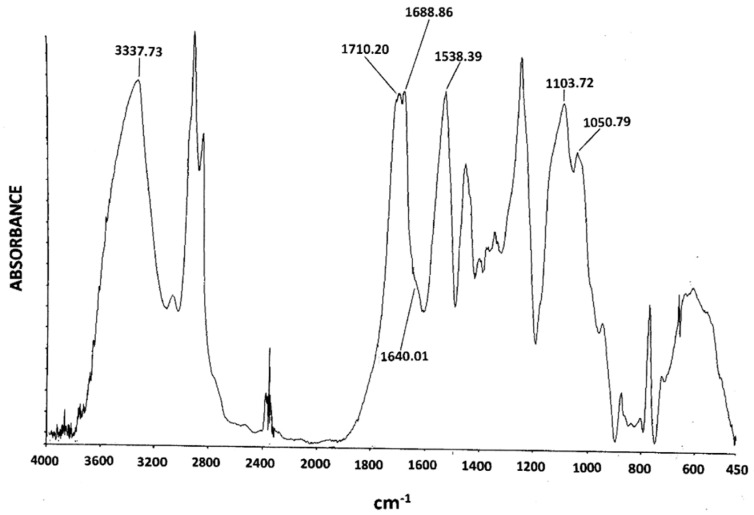
FTIR spectrum of the poly(ether urethane), synthesized with HDI and PEG200 at a molar ratio of 1:1.

The synthesis resulted in a solid, strong, and highly flexible polymer, the weight of which was close to the stoichiometrically calculated weight. Figure 1 exhibits the FTIR spectrum of the segmented polyurethane, synthesized with hexamethylene diisocyanate (HDI) and PEG200 at a molar ratio of 1:1.

Although as seen in Scheme 1, the theoretical polymer repeating unit contains only one type of carbonyl, i.e., the urethane carbonyl, the FTIR spectrum of the synthesized polymer clearly exhibits two adjacent strong and sharp carbonyl-stretching absorbances at around 1710 cm^−1^ and at 1687 cm^−1^ of almost equal intensity. As was also previously demonstrated, the absorbance at around 1710 cm^−1^ belongs to the carbonyl-stretching vibrations of the urethane group [26,27], though slightly shifted to the right as compared with the urethane carbonyl-stretching absorbance previously observed at 1717 cm^−1^ [35,36]; this shift most probably occurred due to hydrogen bonding. This absorbance (Figure 1), though, also appears to exhibit a slight shoulder or thickening to the left at around 1717 cm^−1^, which may be due to non-hydrogen-bonded urethane groups.

The absorbance at 1688 cm^−1^ belongs to the carbonyl-stretching vibrations of tertiary oligo-uret structures, as also previously demonstrated [35,36,37,38]. As demonstrated previously [35,36,37,38] and as described in the above Introduction section, the tertiary oligo-uret structures are the result of the further side reaction of already formed secondary nitrogens during synthesis with an isocyanate, thus creating a tertiary nitrogen and a newly formed NH group—which, in turn, can similarly further side-react with an additional isocyanate, again creating a tertiary nitrogen and a new NH group, and so on. Each additional consecutive side reaction increases the array of electron-withdrawing groups preceding the terminal NH in the structure—increasing the polarity of the H atom on the said NH group, thereby also increasing its reactivity toward an isocyanate group. Hence, the reaction exhibits the preferential predominant formation of the tertiary oligo-uret network structures [35,36,37,38]. The urethane carbonyl-stretching absorbance is located at a higher wavenumber than that of the tertiary oligo-uret structures, which is consistent with its close proximity to the more electronegative oxygen atom in the urethane group.

A small shoulder can be observed to the right of this peak, at around 1640 cm^−1^. An absorbance at this location was previously demonstrated in diisocyanate-derived polyureas, as the stretching absorbance of carbonyls in biuret structures [37,38]. As two-sided biurets are actually short tertiary oligo-uret structures, in the present polymer, this absorbance most probably belongs to carbonyls in short tertiary oligo-uret structures that constitute short branches of larger tertiary oligo-uret structures and/or branches of secondary tertiary oligo-uret crosslinkers of the larger tertiary oligo-uret network structures, which, in turn, crosslink the main polymer chains.

The very strong absorbance at 1103 cm^−1^ belongs to the C–O–C stretching vibrations of the PEG ether groups. It is important to also take note of the strong and sharp adjacent absorbance at 1050 cm^−1^ (Figure 1). This absorbance (as a shoulder to the right of the main C–O–C stretching absorbance) is highly characteristic of PEG in crystalline form (and is totally absent in amorphous PEG) [39]. This strong indication that the PEG segments in the polymer are in a crystalline state is highly surprising, since PEG200 itself is an amorphous liquid that does not crystallize under any conditions (PEG crystallization at room temperature occurs at and above MW = 1000). 

Additional absorbances in the spectrum (Figure 1) are as follows: the strong and sharp absorbance at 1538 cm^−1^ due to the C–N–H deformation; the strong N–H stretching absorbance at 3337 cm^−1^; the symmetric and antisymmetric N–C–N stretching vibrations at 1460 cm^−1^ and 1261 cm^−1^; and the characteristic methylene group absorbances, i.e., the symmetric and antisymmetric CH stretching vibrations at 2851 cm^−1^ and 2953 cm^−1^; the CH bending vibrations at 1450 cm^−1^; and the CH rocking vibrations at 616 cm^−1^. It is also interesting to note that the spectrum exhibits a very much increased intensity of the asymmetric CH stretching absorbance at 2953 cm^−1^, as compared to the symmetric CH stretching absorbance at 2851 cm^−1^, which is highly consistent with a very significant chain alignment in this polymer, with a most probable consequent parallel extended-chain interaction, thus preferentially enabling the asymmetric CH-stretching vibration, and less so enabling the symmetric CH-stretching vibration—being relatively hindered by the said parallel inter-chain interaction.

It is also important to take note of the almost complete absence of the N=C=O absorbance at 2270 cm^−1^, indicating that all the isocyanate groups in the system have reacted.

**Scheme 2:**
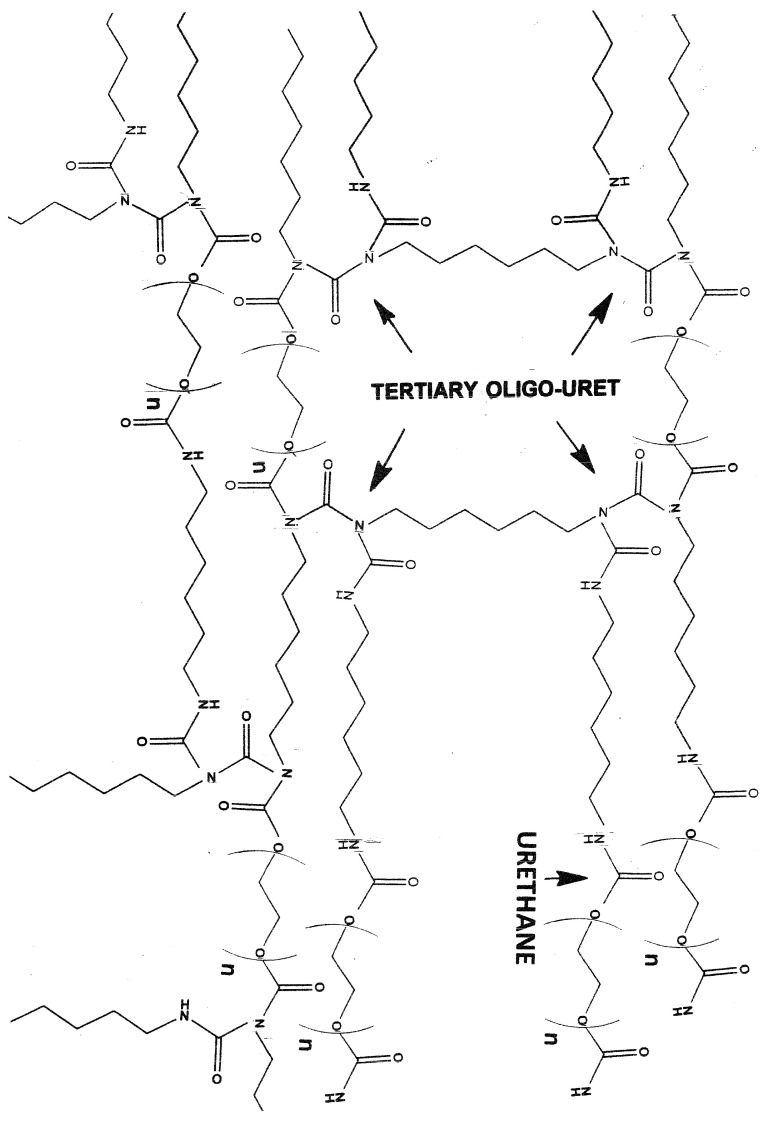
Schematic representation of the poly(ether urethane) chemical structure, exhibiting side-reacted crosslinking networks containing both urethane and tertiary oligo-uret chemical structures. Each NH group in the scheme may further react with an isocyanate group, further resulting in hierarchical tertiary oligo-uret structures of increased length and complexity.

Another important parameter that may be observed in the FTIR spectrum of the polymer (Figure 1) is the very pronounced width of the N–H stretching absorbance at 3337 cm^−1^. It was recently demonstrated for polyurea synthesized via the water–diisocyanate synthesis pathway [38] that the much enhanced width of this absorbance is not due to effects of hydrogen bonding, but it occurs as a consequence of this peak consisting of a relatively wide distribution of N–H stretching absorbance locations, stemming from the wide length distribution of the tertiary oligo-uret hierarchical network structures in the polymer. With an increasing length of the tertiary oligo-uret structure and an increasing number of tertiary nitrogens and carbonyls which are situated between the two terminal NH groups, the electron-withdrawing activity of the environment is increased, thus lowering the NH groups’ electron density (Scheme 2). This, in turn, leads to a wide distribution of the FTIR NH-stretching vibration locations, as a function of the various tertiary oligo-uret lengths in the polymer and of the additional location for the NH groups in the non-side-reacted urethane structures [38]. This is in strong contrast with the extremely thin and sharp N–H stretching absorbance peak in a polymer exclusively containing biuret structures [37]. This is due to the fact that all biuret structures are exactly the same and exhibit a symmetrical structure. This was further confirmed through the synthesis of a new polymer, inherently containing oligo-uret structures in the polymer repeating unit, leading to a very high content of tertiary oligo-uret structures following further side reactions. Consequently, several separate sharp N–H stretching absorbances with a common, very wide base were present in the FTIR spectrum of this polymer, which confirmed the above-described interpretation [38]. 

**Figure 2:**
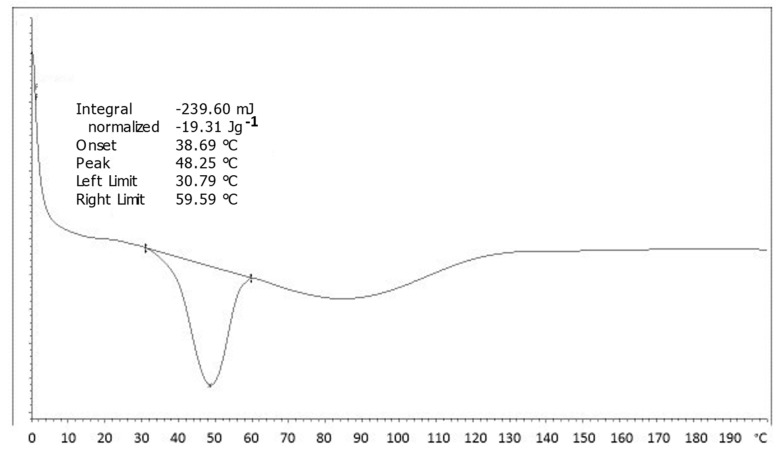
Differential scanning calorimetry (DSC) thermogram of the poly(ether urethane) synthesized with HDI and PEG200 at a molar ratio of 1:1.

Figure 2 exhibits the differential scanning calorimetry (DSC) analysis of the resulting polymer. A very significant endotherm appears at 48.25 °C (ΔH = 19.31 J/g), which is characteristic of PEG melting [39]. This may indicate the possibility that the PEG200 soft segments in the polymer have crystallized, which is in strong agreement with the observed strong and sharp FTIR absorbance (at 1050 cm^−1^) to the right of the main C–O–C stretching absorbance, which is highly characteristic of PEG in crystalline form [39] (Figure 1). In the crystalline state, the FTIR spectrum of PEG is, in some aspects, different from that in the molten state, which may be explained by the fact that in the crystalline state, the chain conformation consists of helical turns, with internal turns around the O–CH_2_, CH_2_–CH_2_, and CH_2_–O bonds of trans, gauche, and trans, respectively [39]. In the molten state, the conformation becomes disordered [39]. 

The theoretical melting enthalpy for 100% crystalline PEG is ΔHm^0^ = 196.8 J/g [40]. Thus, the degree of crystallinity of the PEG200 soft segments in the present polymer may be calculated by relating the melting endotherm enthalpy hereby obtained (ΔH = 19.31 J/g) to the ΔHm^0^ of PEG and normalizing it to the fraction of the PEG segments in the polymer, which accounts for a PEG soft segment degree of crystallinity of 18% (i.e., 18% of the soft segments in the polymer have crystallized).

Again, this is highly surprising, since the PEG of MW = 200 in its pure unreacted form is an amorphous liquid that does not crystallize under any conditions; nevertheless, the data obtained suggest the possible presence of crystalline domains mainly consisting of PEG chains.

The very wide and shallow endotherm at around 90 °C is due to the evaporation of the hygroscopic water molecules, which were adsorbed from the atmosphere by the relatively hydrophilic polymer.

Polymers typically crystallize via two possible crystallization mechanisms: 1. The extended-chain mechanism, in which the polymer chains are stretched and oriented in parallel during the crystallization process; 2. The chain-folding mechanism, in which the polymer chains repeatedly fold during the crystallization process to form lamellar crystalline structures [41,42,43,44,45,46,47]. The crystallization of polymers via the extended-chain mechanism can only occur when a very significant stretching force is applied on the polymer during the crystallization process—as commonly applied during the industrial production of polymeric fibers. Otherwise, polymers naturally crystallize via the chain-folding mechanism [41,42,43,44,45,46,47]. 

The molecular weight of PEG200 is much lower than the crystallization threshold for PEG, and, also, the very short chain length at this molecular weight does not offer the possibility of a chain-folding mechanism. Still, the above-described results indicate a significant crystallization of the PEG200 soft segments in the polymer. The only possible mechanism of PEG200 soft segment crystallization that most probably occurred here is the parallel extended-chain orientation of the PEG segments, anchored in place by the crosslinking structures at their extremities, in close-enough parallel proximity to enable crystallization. The parallel orientation of the chains most probably occurred both before and during the crosslinking process, due to the significant shear forces induced through the mechanical stirring of the reactor content during synthesis.

The co-crystallization of urethane groups along with the PEG200 segments may be ruled out due to the highly pronounced differences between these two chemical structures, as reflected in the following characteristics: pronounced different polarities favoring microphase separation; the significant planar structure and rigidity of urethane groups versus the very pronounced flexibility of the PEG ether groups; and the fact that a large portion of the initially formed urethane groups are side-reacted and held within crosslinking network structures.

Although hydrogen bonding most probably does occur in at least some of the non-side-reacted urethanes, these very short hard segments consisting of only two urethanes, separated by six methylenes, also most probably do not separately crystallize. Furthermore, a well-known phenomenon occurring in segmented polymers (i.e., in block copolymers) is inter-segmental interference, in which the crystallization of a segment of more dominant physical properties (i.e., of a hard segment) diminishes and even prevents the crystallization of the adjacent soft segments covalently bonded thereto [48,49]. This was also demonstrated in very recent research [27] showing that the crystallization of the hard segments in the synthesized polyurethanes prevented the crystallization of the polycaprolactone (PCL) soft segments of these polymers.

Thus, any occurrence of urethane hard segment crystallization in the present polymer would have definitely prevented the crystallization of the hereby very short PEG200 soft segments.

Scheme 2 exhibits a possible schematic representation of the molecular structure of the polymer. Each N–H group in the structure can potentially further side-react with an isocyanate group, creating a tertiary nitrogen and a new secondary nitrogen, which may in turn react with an isocyanate, and so on, creating increasingly complex hierarchical crosslinking networks. Crosslinking may occur not only between main polymer chains but also within and between the crosslinking network structures. It may be observed that although the tertiary oligo-uret structures are highly complex, they offer the possibility of the formation of the close and parallel alignment of groups of polymer chains in a crosslinked state—which is in strong agreement with the extended-chain crystallization mechanism described above. 

**Figure 3:**
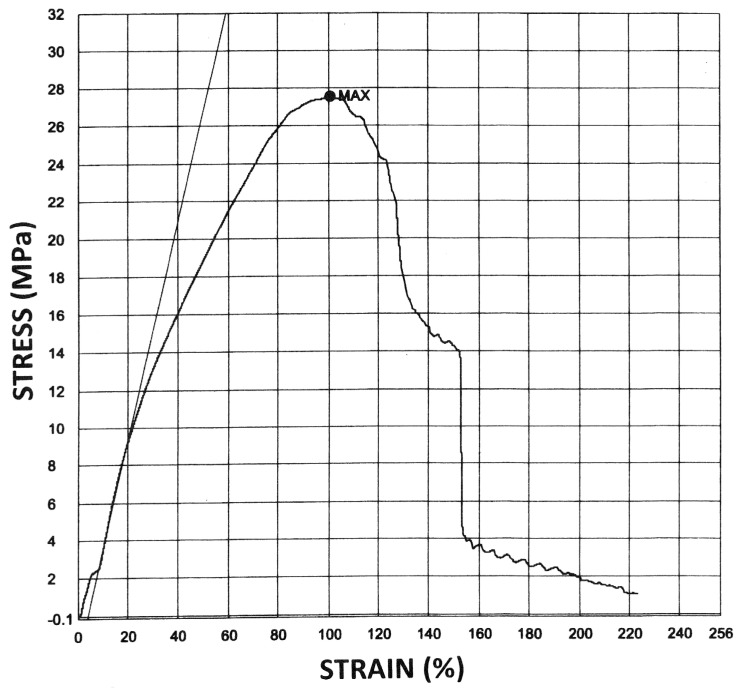
Representative tensile stress–strain curve of the poly(ether urethane), synthesized with HDI and PEG200 at a molar ratio of 1:1 (the tensile mechanical properties of all the measurements performed, including average and standard deviation, are exhibited in Table 1 below).

Figure 3 exhibits the tensile mechanical properties of a representative tested sample of the segmented polyurethane synthesized with hexamethylene diisocyanate (HDI) and PEG200 at a molar ratio of 1:1. The polymer exhibits a significantly high tensile strength of close to 28 MPa (the average tensile strength of the seven samples tested in the present research was 28.8 MPa, with a standard deviation of 3.6—as detailed in Table 1). By comparison, the tensile strengths of previously reported segmented polyurethanes, synthesized using the same diisocyanate (HDI) but with much longer hard segments (consisting of two urethanes and two amides and, additionally, containing a C=C double bond) and a PTMG2000 soft segment [11] or a polybutadiene2000 soft segment [12], were 50 MPa and 25 MPa, respectively. Additional examples of polyurethane elastomer tensile properties reported in the literature are as follows: polyurethane elastomers consisting of PTMG soft segments, MDI, and a butanediol (BD) chain extender exhibited tensile strengths of between 20 and 45 MPa and exhibiting elongations of between 650 and 1100% [50,51,52]; poly (urethane urea) elastomers containing various soft segments consisting of polyethers, or polyesters such as polycaprolactone, exhibited tensile strengths of between 10 and 38 MPa and exhibiting elongations of between 100 and 360% [53,54,55,56,57]. The presently designed polyurethane is within the range between these tensile strength values of polyurethane elastomers, despite the very short hard segment of the present polymer. Nevertheless, as an extremely wide range of chemical structures can be designed and exist in the vastly large and versatile family of polyurethanes, the Young’s moduli of polyurethanes range from 1 to 1000 MPa [57], and typical elongations at break are typically higher than 200% [50,51,52,53,54,55,56,57,58]. The relatively low elongation at break obtained in the present poly(ether urethane) is due to and highly consistent with the above-discussed pre-aligned state of the short polymer soft segments, the stretching of which being the main source of the significantly high elongations of polyurethane elastomers. It is, therefore, hereby suggested that the main source of the elongation in the present polymer is most probably the flexibility of the significantly large crosslinking hierarchical tertiary oligo-uret network structures, resulting from the above-discussed side reactions.

The industrial synthesis of the polymer in appropriately shaped molds may provide further optimization, which will most probably lead to even higher tensile strength. A well-known and commonly used industrial processing method for crosslinked polyurethanes and poly(urethane urea)s is reaction injection molding (RIM) [59,60]. Nevertheless, the occurrence of some flaws, imperfections, and internal stresses in the final product polymer is practically inevitable, even in highly optimized industrial injection molding processes [61]. It is also important to take note of the occurrence of a controlled failure mechanism (occurring gradually) as opposed to catastrophic failure (which occurs abruptly/instantly). This is highly advantageous in terms of mechanical properties, as it results in a relatively high breaking energy (as reflected in the relatively large area beneath the curve) and, thus, a relatively high polymer toughness. A relatively high modulus of 58.2 MPa (Figure 3) (average of seven measurements 110.2 MPa—Table 1) was obtained, along with a significantly steep ascending first linear part of the stress–strain curve. This is in contrast with the typically encountered elastomer tensile stress–strain curve, which commonly exhibits a very shallow initial ascent (and a very low modulus), which is then followed by a very steep ascent as the soft segments reach a high degree of parallel alignment in the direction of the applied tensile stress [11,12,50,51,52,53,54,55,56,57,58]. The fact that the present stress–strain curve (Figure 3) immediately starts with a steep linear ascent and lacks the usually encountered initial shallow ascent is due to the fact that in the present polymer, the soft segments are already aligned and extended—as discussed above.

**Figure 4:**
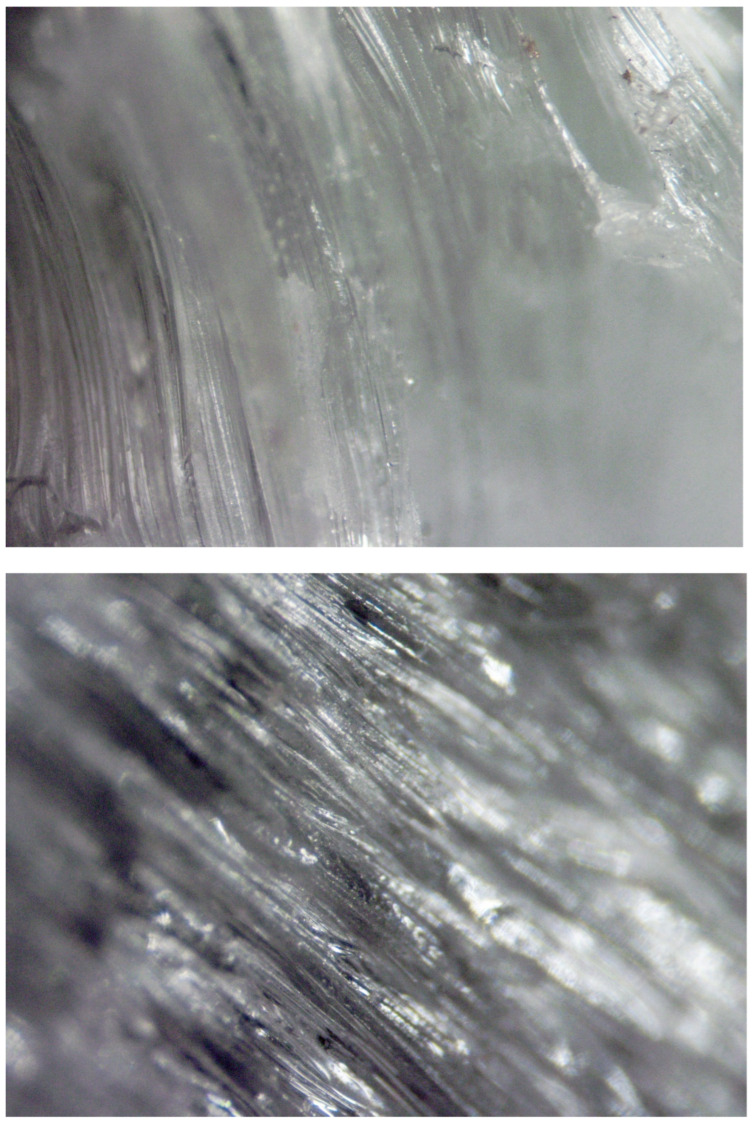
Optical microscopy photographs of the poly(ether urethane) synthesized with HDI and PEG200 at a molar ratio of 1:1, (magnification ×50). Upper photograph—the surface is indicative of very significant polymer orientation; lower photograph—exhibits a representative occasionally-occurring fibrillation region and the occasional formation of some inter-fibrillar gaps within the fibrillation regions, due to the very significant chain orientation within the polymer.

Figure 4 exhibits optical microscopy photographs of the surface of the poly(ether urethane) synthesized with HDI and PEG200 at a molar ratio of 1:1. The optical microscopy images are indicative of the very significant chain orientation occurring, which occasionally even results in regions of fibrillation and occasionally occurring inter-fibrillar gaps within these regions, as commonly observed in polymeric fibers. This is highly consistent with and confirms the above-discussed soft segments orientation and their possible extended-chain crystallization mechanism.

**Figure 5:**
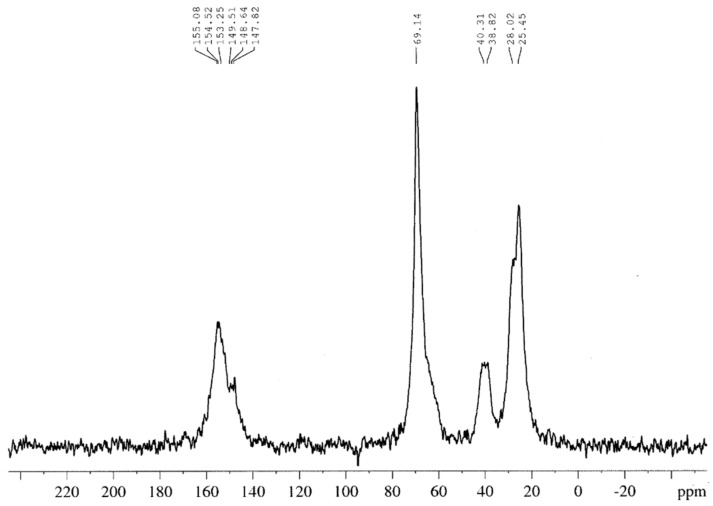
Solid-state ^13^C NMR spectrum of the poly(ether urethane), synthesized with HDI and PEG200 at a molar ratio of 1:1.

Figure 5 exhibits the solid-state ^13^C NMR spectrum of the segmented polyurethane, synthesized with hexamethylene diisocyanate (HDI) and PEG200 at a molar ratio of 1:1. Two very close resonances at 25 ppm and at 28 ppm belong to the carbons of the four inner methylene groups of the HDI hexamethylene group (the two central methylenes resonate at 25 ppm and the following two methylenes at 28 ppm). The resonance at around 40 ppm is attributed to the outermost two methylenes of the HDI hexamethylene group, bonded to the nitrogen atoms. The very strong and sharp resonance at 69 ppm belongs to the two PEG methylenes, bonded to the ether oxygen atoms. The very high intensity of this resonance stems from the high abundance of these groups originating from the PEG200 soft segments. This resonance exhibits a relatively low-intensity shoulder at around 64 ppm, belonging to the two outermost methylenes of the PEG segments which are bonded to the urethane (or urethyl-in allophanate and tertiary oligo-uret structures) ether oxygen atom.

A strong carbonyl carbon resonance appears at 155 ppm, exhibiting another very significant shoulder resonance to the right at 148 ppm. The strong resonance at 155 ppm significantly widens to the left, indicating a strong concomitant adjacent presence of the characteristic resonance of carbonyls in tertiary oligo-uret structures at 158 ppm [37,38]. This indicates that at least four types of carbonyls are present in the polymer structure. The tertiary oligo-uret carbonyl was previously identified in polyureas to resonate at 158 ppm [37,38]. The urethane carbonyl was previously demonstrated to resonate at 155 ppm [36]. These two resonances in the spectrum (Figure 5) are of almost equal intensity (and strongly overlapping due to their close proximity), which is in strong agreement with the almost equal intensities of the carbonyl-stretching absorbances of these two structure types in the FTIR spectrum of the same polymer (Figure 1). Nevertheless, the very wide common base of these carbonyl resonances inevitably indicates that these very wide resonance peaks are composed of a very wide distribution of carbonyl types, each resonating at a slightly different ppm, due to slightly different electron densities stemming from the different electron-withdrawing environments induced by the vast variety of possible structural configurations in the crosslinked polymer hierarchical network (as schematically represented in Scheme 2). Thus, for example, the tertiary oligo-uret structures may be of a variety of different lengths, exhibiting an increasing electron-withdrawing environment for each carbonyl with the increasing length of a specific structure. As also seen in Scheme 2, the tertiary oligo-uret structures can include PEG soft segments in the structure or can alternatively form without including the PEG segments in different regions of the crosslinking networks. The adjacent presence or absence of the ether oxygens of the PEG soft segments may also influence the electron-withdrawing environment and, thus, the electron densities of some of the carbonyls in a specific structure. Also, relatively short tertiary oligo-uret structures may form as secondary crosslinkers of larger tertiary oligo-uret network structures that, in turn, crosslink the main polymer chains (as seen in Scheme 2). The resonance shoulder at 148 ppm may be attributed to the carbonyls of these short tertiary oligo-uret secondary crosslinking structures, exhibiting a less electron-withdrawing environment and, thus, a higher electron density of the carbonyls within these structures. These, and many more possible structural network variations, most probably contributed to the occurrence of the very large width of the carbonyl carbon resonances in the present NMR spectrum.

An additional poly(ether urethane) was hereby synthesized, using a higher molecular weight of PEG (i.e., PEG400) under the same conditions and the same molar ratio with HDI. The synthesis resulted in a wax-like material, without any significant properties. This is most probably due to the fact that the molecular weight of the PEG soft segment is hereby significantly increased (doubled), while the very short, hard segment length (of only two urethane groups) remains constant, and the distances between them concomitantly increase, i.e., they occur at a significantly decreased frequency along the polymer chain. This also increases the distance between side reaction sites, which also most probably decreases the efficacy of secondary crosslinking between the more distanced tertiary oligo-uret network components.

**Figure 6:**
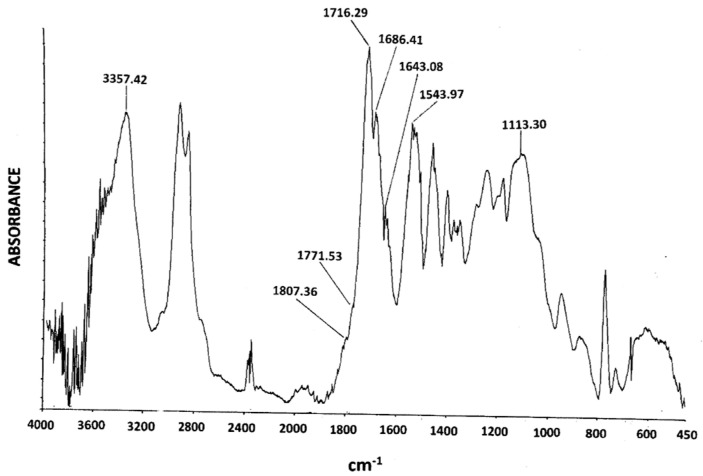
FTIR spectrum of the poly(ether urethane), synthesized with HDI and PEG400 at a molar ratio of 1:1.

Figure 6 exhibits the FTIR spectrum of the segmented polyurethane, synthesized with hexamethylene diisocyanate (HDI) and PEG400 at a molar ratio of 1:1.

A very strong and sharp carbonyl-stretching absorbance is observed at 1716 cm^−1^, belonging to the urethane groups. An additional sharp but significantly lower-intensity carbonyl-stretching absorbance appears at 1686 cm^−1^, belonging to the formed tertiary oligo-uret network structures. The significantly lower intensity of this absorbance as compared to the urethane carbonyl absorbance and as compared to the same absorbance in the polyurethane synthesized with PEG200 (Figure 1) indicates a much lower efficacy of tertiary oligo-uret network structures formation in the polyurethane synthesized with PEG400, which is consistent with the very poor properties of the resulting polymer. It is also important to notice the sharp carbonyl-stretching absorbance at 1643 cm^−1^, which is now of significant intensity (as compared to the small shoulder around this region in the spectrum of the polyurethane synthesized with PEG200, in Figure 1). This is most probably due to a significantly increased amount of relatively short tertiary oligo-uret branching—which did not result in final fully developed secondary crosslinking networks, due to the much greater distance between side reaction sites resulting from the hereby much longer PEG400 soft segments. Two additional small carbonyl-stretching shoulders at 1807 cm^−1^ and at 1771 cm^−1^ are due to the presence of a small amount of non-further-reacted allophanate groups, again indicating a less complete tertiary oligo-uret network structure formation in this polymer, as compared with the polyurethane synthesized with PEG200, where residual allophanate groups are not observed (Figure 1).

**Figure 7:**
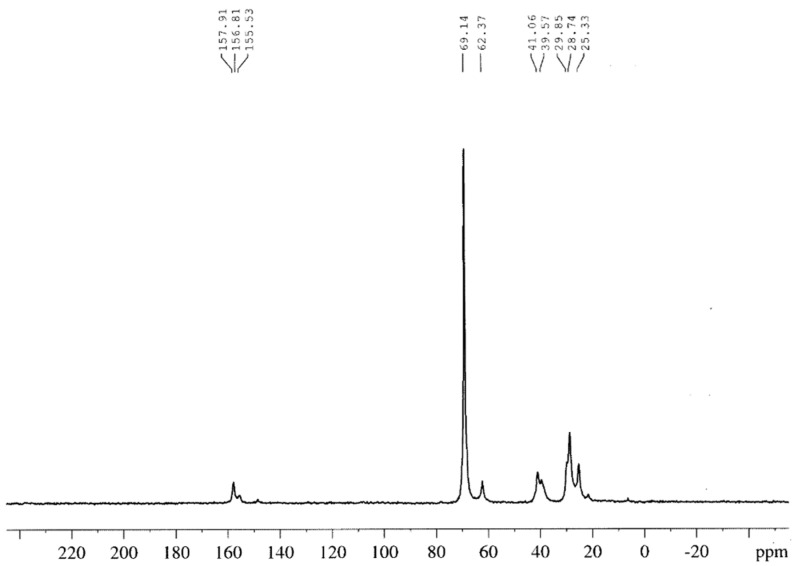
Solid-state ^13^C NMR spectrum of the poly(ether urethane), synthesized with HDI and PEG400 at a molar ratio of 1:1.

Figure 7 exhibits the solid-state ^13^C NMR spectrum of the poly(ether urethane), synthesized with HDI and PEG400 at a molar ratio of 1:1. Two resonances at 25 ppm and at 28 ppm belong to the carbons of the four inner methylene groups of the HDI hexamethylene group (the two central methylenes resonate at 25 ppm and the following two methylenes at 28 ppm). The split resonances at 39 ppm and 41 ppm are attributed to the outermost two methylenes of the HDI hexamethylene group, bonded to the nitrogen atoms (which are split since they are bonded to either a secondary nitrogen, or a side-reacted tertiary nitrogen). The very strong and sharp resonance at 69 ppm belongs to the two PEG400 methylenes, bonded to the ether oxygen atoms. The very high intensity of this resonance stems from the very high abundance of these groups originating from the PEG400 soft segments. The very small resonance at around 62 ppm belongs to the two outermost methylenes of the PEG400 segments which are bonded to the urethane (or urethyl—in allophanate and tertiary oligo-uret structures) ether oxygen atom. Two sharp carbonyl carbon resonances are seen at 158 ppm and at 155 ppm, due to the tertiary oligo-uret carbonyls and the urethane carbonyls, respectively. The very small resonance shoulder at 162 ppm belongs to the allophanate urethyl carbonyl carbon, indicating the presence of a small amount of allophanate groups in the polymer that did not further side-react to form tertiary oligo-uret structures. This is in agreement with the two small carbonyl-stretching shoulders at 1807 cm^−1^ and at 1771 cm^−1^ observed in the FTIR spectrum of the same polymer, belonging to a small amount of non-further side-reacted allophanate groups (Figure 6). It is interesting to note the total absence of carbonyl carbon resonance at 148 ppm, belonging to the shorter secondary tertiary oligo-uret crosslinkers of larger tertiary oligo-uret network structures that, in turn, crosslink the main polymer chains which strongly appeared in the solid-state ^13^C NMR spectrum of the poly(ether urethane) synthesized with HDI and PEG200 (Figure 5). This is also in agreement with the observed less efficient tertiary oligo-uret formation in the FTIR spectrum of the same polymer and the strong appearance of the carbonyl stretching absorbance at 1641 cm^−1^, most probably belonging to the short non-crosslinking tertiary oligo-uret branches (Figure 6), which is also highly consistent with the very poor physical properties of this polymer.

**Figure 8:**
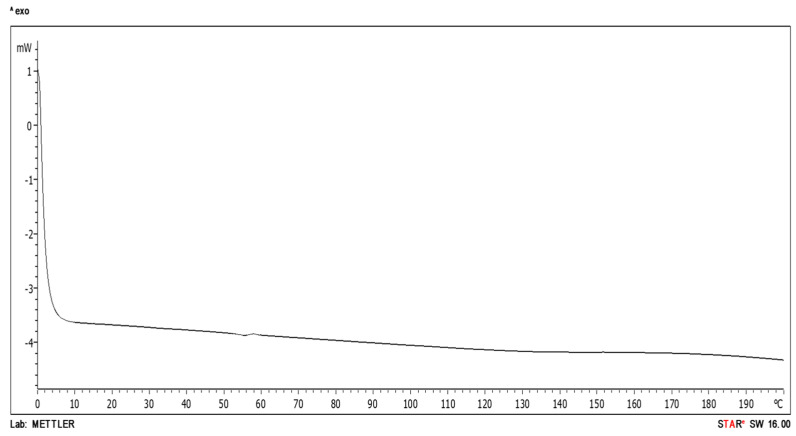
Differential scanning calorimetry (DSC) thermogram of the poly(ether urethane) synthesized with HDI and PEG400 at a molar ratio of 1:1.

Figure 8 exhibits the DSC analysis thermogram of the poly(ether urethane) synthesized with HDI and PEG400 at a molar ratio of 1:1. The polymer is essentially amorphous, exhibiting no significant melting endotherm (by looking closely, though, a very small, almost imperceptible endotherm may be observed at around 53 °C, indicating that maybe a very small portion of the chains may have crystallized). This is in strong contrast with the above polymer synthesized with HDI and PEG200, which exhibited a very significant melting endotherm at a temperature characteristic of PEG melting [39] (Figure 2). This amorphous state is most probably due to the above-described less efficient formation of the tertiary oligo-uret networks and their less efficient secondary crosslinking—thus most probably not efficiently stabilizing the soft segments in an aligned configuration. 

**Figure 9:**
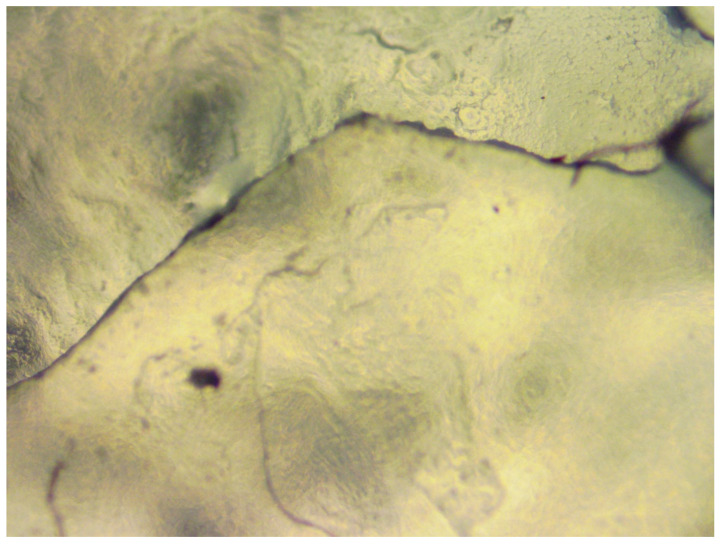
Optical microscopy photograph of the poly(ether urethane) synthesized with HDI and PEG400 at a molar ratio of 1:1, (magnification ×50). The surface is not indicative of any significant polymer chain orientation. Occasionally occurring micro-cracks are strongly visible.

Figure 9 exhibits optical microscopy photograph of the surface of the poly(ether urethane) synthesized with HDI and PEG400 at a molar ratio of 1:1. The polymer images are not indicative of any significant chain orientation in the polymer, which is also in agreement with the amorphous state of the polymer. This is in strong contrast with the highly oriented and even fibrillar structure image of the polymer synthesized with HDI and PEG200 under the same magnification. It is also important to take note of the occasional appearance of micro-cracks in the polymer, which are also most probably the source of the very poor mechanical properties of this polymer.

Due to the poor mechanical properties of this polymer and the inherently present micro-cracks, the polymer broke either during sample preparation or during the fastening of the machine grips on the samples—thus, the measurement of the mechanical properties of this polymer was not possible. 

In order to further confirm and elaborate the preferential occurrence of the above-described side reactions and of the resulting tertiary oligo-uret network formation, a completely new type of synthesis was hereby designed and performed, this time reacting the diisocyanate (HDI) with a mono-hydroxy-terminated PEG, namely with PEG350 monomethyl ether (PEG350M) (instead of the diol-terminated PEG used in the synthesis described above).

The synthesis was performed under exactly the same conditions as the above-described synthesis, and at a HDI–PEG350M molar ratio of 1:1. Consequently, there are two N=C=O groups for each OH group in the reaction system.

Thus, one of the following possibilities may hypothetically occur as a result of the synthesis:

In the case that no (or very few) side reactions will occur, only trimers will be formed, and only urethane groups will be present in the final product, along with a large excess amount of unreacted isocyanate groups.

In the case that a significant amount of urethane groups remain non-side-reacted—and the remaining excess of free N=C=O groups in the system preferentially further side-react with the significantly smaller amount of already formed allophanate secondary uret groups to form increasingly longer tertiary oligo-uret structures, instead of forming additional new allophanates with the remaining urethane groups—this will conclusively support the above-suggested energetically favorable mechanism for the preferential occurrence of the consecutive side reactions. In this case, the resulting polymer will be a hyper-branched structure, consisting of relatively long PEG350 branches one-sidedly connected to tertiary oligo-uret hierarchical network structures.

**Figure 10:**
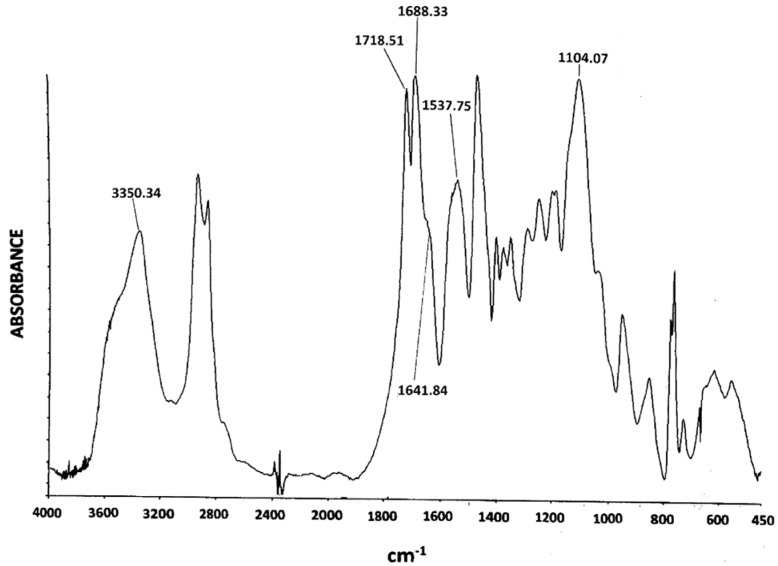
FTIR spectrum of the hyper-branched polymer, synthesized with HDI and PEG350M at a molar ratio of 1:1.

The synthesis resulted in a soft amorphous polymer. Figure 10 exhibits the FTIR spectrum of the resulting polymer, hereby synthesized with PEG350M and HDI at a 1:1 molar ratio.

The almost complete absence of an absorbance peak at 2270 cm^−1^ indicates that practically all the isocyanate groups in the system have reacted. Two very strong and sharp carbonyl-stretching absorbances are present in the spectrum—the absorbance at 1717 cm^−1^ belongs to the urethane carbonyl; and the higher-intensity absorbance at 1687 cm^−1^ belongs to the carbonyls in tertiary oligo-uret structures. A shoulder to the right of this peak, at around 1640 cm^−1^, may be clearly observed. As also described above, an absorbance at this location was previously demonstrated in diisocyanate-derived polyureas, as the stretching absorbance of carbonyls in biuret structures [37,38]. As two-sided biurets are actually short tertiary oligo-uret structures, in the present polymer, this absorbance most probably belongs to carbonyls in relatively short tertiary oligo-uret branching—which did not result in final fully developed secondary crosslinking networks. This absorbance shoulder is of a higher intensity than the shoulder at around the same location in the FTIR spectrum of the polymer synthesized with PEG200 (Figure 1). This most probably stems from some steric interference of the relatively long PEG350 hyper-branching with the efficacy of the tertiary oligo-uret secondary crosslinking structures development. Nevertheless, this absorbance is of a much lower intensity than in the FTIR spectrum of the polymer synthesized with PEG400 (Figure 6), as also explained above.

The absorbance at 1537 cm^−1^ belongs to the C–N–H deformation vibrations. It is interesting to note that the intensity of this absorbance is much lower in the present spectrum (Figure 5) than the same absorbance peak in the spectrum of the polymer synthesized with PEG200 (Figure 1). This strongly indicates a predominantly higher abundance of tertiary nitrogens in the present polymer than in the polymer synthesized with PEG200.

The absorbance at 3350 cm^−1^ belongs to the N–H stretching vibrations of the urethane groups, as well as of the two terminal N–H groups at the extremities of each tertiary oligo-uret structure (Scheme 2). The extremely great width of this peak strongly indicates the presence of a wide distribution of lengths and hierarchical configurations of the tertiary oligo-uret structures, as recently demonstrated [29] and as described above. This absorbance (at 3350 cm^−1^), however, is of a much lower intensity in the present spectrum (Figure 5) than in the spectrum of the polymer synthesized with PEG200 (Figure 1). This, again, strongly indicates a predominantly higher abundance of tertiary nitrogens in the present polymer than in the polymer synthesized with PEG200.

As the amount of methylene groups is significantly increased in the present hyper-branched polymer as compared to the polymer synthesized with PEG200 due to the significantly higher molecular weight of the PEG350M, the CH-related absorbances could not be used as internal standards for intensity comparison. Thus, the intensities of the above-discussed NH-related absorbances were qualitatively compared to the intensities of the carbonyl stretching absorbances—the significantly lower intensities of the NH-related absorbances in the hereby hyper-branched polymer, than in the polymer synthesized with PEG200, as related to the intensities of the carbonyl-stretching absorbances of the same polymers, respectively, clearly indicates a significantly higher content of tertiary nitrogens (and a significantly lower NH content) and, thus, a significantly higher content of tertiary oligo-uret structures in the PEG350M-containing polymer.

The strong absorbance at 1104 cm^−1^ belongs to the C–O–C stretching vibrations of the PEG350M ether groups. It is important to also note the complete absence of the adjacent peak at 1050 cm^−1^ in the present spectrum (Figure 10)—this peak was strongly present in the spectrum of the polymer synthesized with PEG200 (Figure 1) which is characteristic of PEG in crystalline form [39]. The complete absence of this peak in the FTIR spectrum (Figure 8) is in strong agreement with the amorphous nature of the present polymer, synthesized with PEG350M. This is also highly consistent with the fact that the present polymer is a hyper-branched polymer, the significantly long PEG350 branches of which sterically inhibit any close intermolecular or intersegmental interactions. Also, in the present polymer, the PEG branches are not oriented (extended) since they are only chemically anchored at one end—the other end being free—and are, thus, most probably in a relatively disordered configuration. This is also reflected in the location of the urethane carbonyl-stretching absorbance in the FTIR spectrum of the present polymer, at 1717 cm^−1^ (Figure 10), as compared to its location in the spectrum of the polymer synthesized with PEG200, at 1710 cm^−1^ (Figure 1). The slight shift to the right (i.e., to a slightly lower wavenumber) of the urethane carbonyl (Figure 1) most probably stems from some hydrogen bonding of these groups, which may be sterically possible in the non-side-reacted urethanes that are chemically bonded to the extended and parallel-oriented crystallized PEG200 soft segments (Scheme 2). In the present hyper-branched polymer, the occurrence of such hydrogen bonds is not possible due to the steric hindrance of the long PEG350 branches.

**Figure 11:**
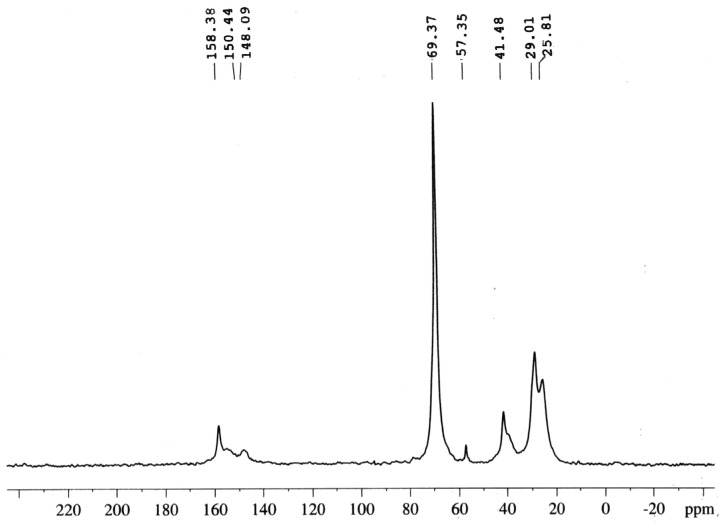
Solid-state ^13^C NMR spectrum of the hyper-branched polymer, synthesized with HDI and PEG350M at a molar ratio of 1:1.

Figure 11 exhibits the solid-state ^13^C NMR spectrum of the polymer hereby synthesized with PEG350M and HDI at a 1:1 molar ratio. 

The two very close, partially overlapping resonances at 25 ppm and at 29 ppm belong to the carbons of the four inner methylene groups of the HDI hexamethylene group (the two central methylenes resonate at 25 ppm and the following two methylenes at 29 ppm). The resonance at around 42 ppm is attributed to the outermost two methylenes of the HDI hexamethylene group, bonded to the nitrogen atoms. This resonance exhibits a shoulder resonance to the right at around 39 ppm. This is a strong indication that these carbons are bonded to two different types of nitrogen atoms, i.e., the tertiary nitrogen and the secondary nitrogen attributed to the 42 ppm and the 39 ppm resonances, respectively. The fact that the resonance at 42 ppm is of a much higher intensity than the resonance at 39 ppm indicates a much higher content of tertiary nitrogens in the polymer than secondary nitrogens. The very small, low-intensity resonance at 57 ppm belongs to the methyl–ether end-group carbon of the PEG350M branches which, as end-groups, are in very low concentration in the polymer. The very strong and sharp resonance at 69 ppm belongs to the two PEG methylenes, bonded to the ether oxygen atoms of the PEG350M branches. The very high intensity of this resonance stems from the high abundance of these groups originating from the PEG350M branches in the polymer.

Four types of carbonyl carbon resonances are clearly present in the spectrum (Figure 11). Among these, the resonance exhibiting the strongest intensity and located at 158 ppm is attributed to the carbonyls in the tertiary oligo-uret structures. This, again, indicates the very high content of carbonyls belonging to tertiary oligo-uret structures in the hyper-branched polymer. The adjacent resonance at 155 ppm belongs to the carbonyls in the non-further-reacted urethane groups of the polymer. This is highly consistent with the above-described assumption that the very wide carbonyl carbon resonance in the NMR spectrum in Figure 5 included both the adjacent resonances, at 155 ppm and at 158 ppm, overlapping and in quasi-equal intensities. The adjacent resonance to the right at 148 ppm, as discussed above, belongs to the relatively short tertiary oligo-uret secondary crosslinkers of larger tertiary oligo-uret network structures (as seen in Scheme 2). The carbonyls of these short tertiary oligo-uret secondary crosslinking structures comprise a less electron-withdrawing environment and, consequently, a higher electron density of the carbonyls within these structures, thus resonating at a lower ppm. A very small resonance shoulder at 162 ppm belongs to the allophanate urethyl carbonyl carbon, indicating the presence of a small amount of allophanate groups in the polymer that did not further side-react to form tertiary oligo-uret structures.

The very high content of tertiary oligo-uret structures in this new polymer, along with the concomitant presence of a significant amount of remaining non-further-reacted urethane groups, indicate and confirm the above-described preferential occurrence of the consecutive side-reactions to form the hierarchical tertiary oligo-uret network structures. These results are also in strong agreement with the results previously obtained by reacting HDI with a mono-amine-terminated molecule (butylamine) at the same molar ratio of 1:1, which also exhibited a predominant and preferential formation of tertiary oligo-uret structures along with a significant amount of remaining non-further-reacted urea groups [28]. Nevertheless, the polymer hereby synthesized with PEG350M resulted in a relatively soft material, due to the very soft and relatively long PEG350M hyper-branching—whereas the polymer previously synthesized by reacting HDI with the mono-amine-terminated butylamine resulted in a highly rigid, rock-hard, hyper-branched polymer, due to the very short butyl branches, and, thus, most probably a much lower free volume and a much tighter, higher degree of crosslinking.

**Scheme 3:**
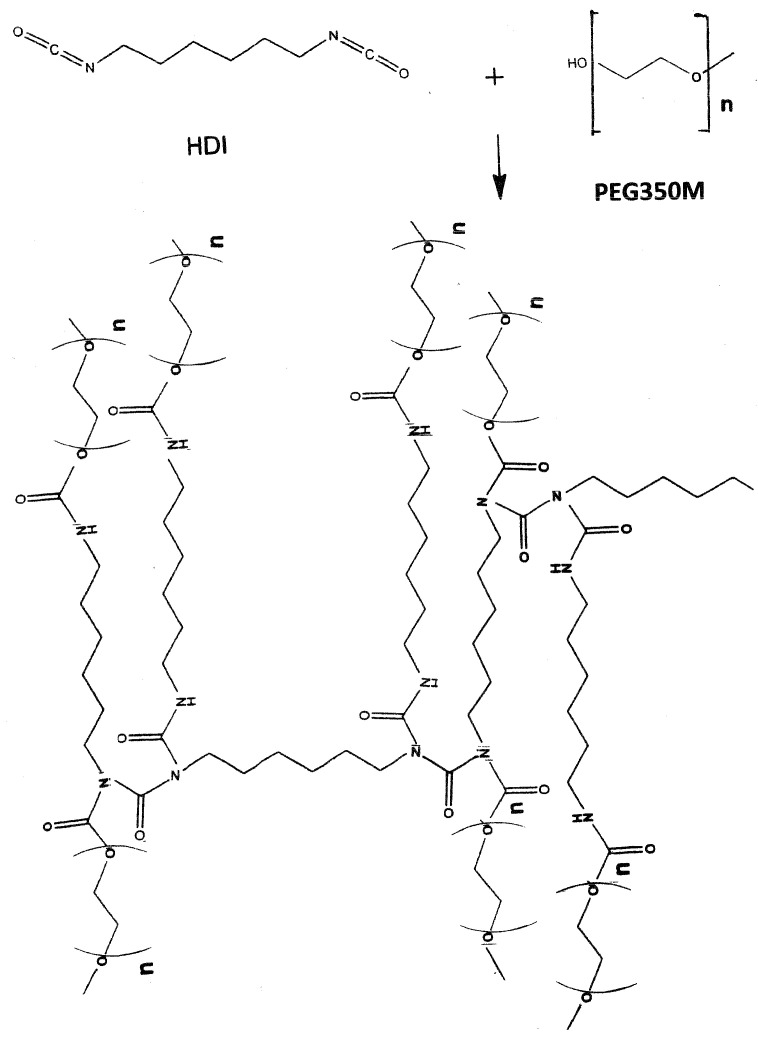
Schematic representation of the molecular structure of the hyper-branched polymer, synthesized with HDI and PEG350M at a molar ratio of 1:1. Each N–H group in the structure can potentially further side-react with an isocyanate group, creating increasingly complex crosslinked hierarchical hyper-branched networks with increasingly numerous PEG350M branches.

Scheme 3 exhibits a possible schematic representation of the molecular structure of the hyper-branched polymer, synthesized with HDI and PEG350M at a molar ratio of 1:1. Each N–H group in the structure can potentially further side-react with an isocyanate group, creating a tertiary nitrogen and a new secondary nitrogen, which may in turn react with an isocyanate, and so on, creating increasingly complex crosslinked hierarchical hyper-branched networks with multiple PEG350M branches. 

The fact that, as demonstrated above, a hyper-branched crosslinked polymer network was obtained by reacting a diisocyanate with a mono-hydroxy-terminated molecule at a molar ratio of 1:1 (thus constituting a 2:1 -N=C=O:OH molar ratio), provides a conclusive proof of the preferential and predominant occurrence of the side-reactions leading to the tertiary oligo-uret network structures formation—and without which, this polymer would have been impossible to obtain. This result is also in strong agreement with a similar hyper-branched crosslinked polymer network previously obtained by reacting a diisocyanate with a mono-amine-terminated reagent at the same molar ratio [37]. 

**Figure 12:**
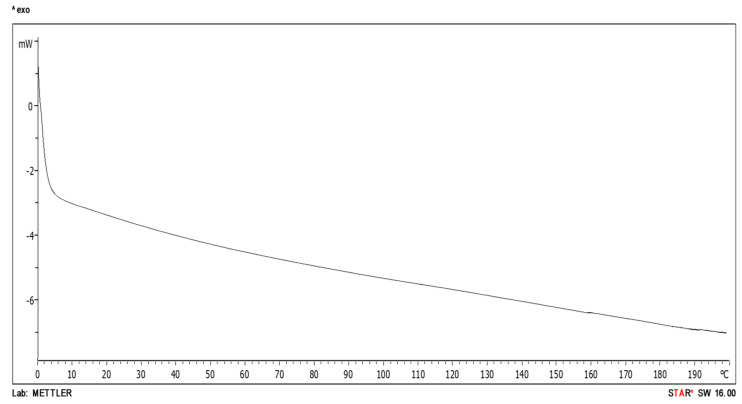
Differential scanning calorimetry (DSC) thermogram of the hyper-branched polymer synthesized with HDI and PEG350M at a molar ratio of 1:1.

Figure 12 exhibits the DSC analysis thermogram of the hyper-branched polymer, synthesized with HDI and PEG350M at a molar ratio of 1:1. As can be clearly observed in the thermogram, the polymer is totally amorphous—which is in complete agreement with the fact that the polymer is highly branched, with significantly long PEG350M branches, thus inhibiting crystallization of this polymer. Nevertheless, the significantly crosslinked tertiary oligo-uret network structure renders the polymer a soft rubbery consistency. 

**Figure 13:**
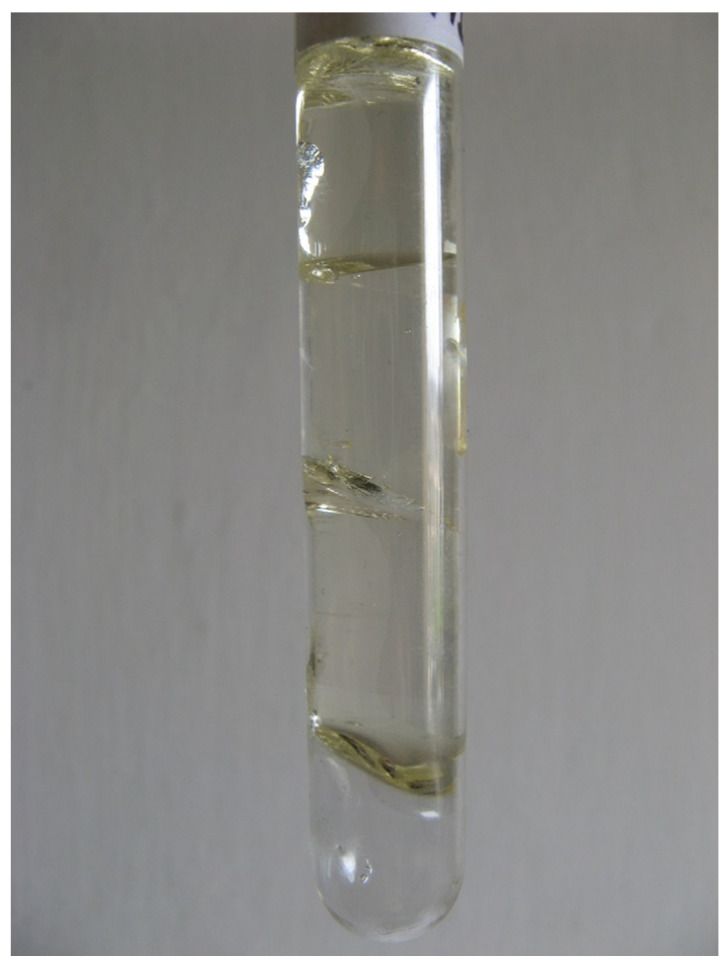
Photograph of a test tube containing the hyper-branched polymer synthesized with HDI and PEG350M at a molar ratio of 1:1. The three V-shaped gaps/cracks observed in the polymer are the result of the significant contraction of the material, as it solidified into a soft rubbery state within the incubation period at room temperature.

Figure 13 exhibits a photograph taken of a test tube containing the hyper-branched polymer synthesized with HDI and PEG350M at a molar ratio of 1:1. The still-hot soft polymer was inserted in test tubes immediately after synthesis, and the sealed test tube was incubated at room temperature and in the dark for at least three months. As observed, the polymer is highly clear and transparent with an apparently very uniform consistency. Nevertheless, some horizontal separation regions are observed along the test tube contents, which are most probably due to the polymer contraction during the incubation period, as the polymer rigidity increased within the incubation period at room temperature. An amount of the still-hot soft polymer was also spread in a large glass Petri dish to form a film; in parallel, an amount of the still-hot soft polymer was spread and pressed between two Kapton sheets—for subsequent mechanical testing. It is interesting to note that these gaps did not occur in the films incubated in the Petri dish or between the Kapton sheets. Nevertheless, the polymer exhibited an extremely strong adherence to both the glass surface and to the Kapton sheets; thus, it could not be non-destructively separated from neither the glass nor the Kapton surface—making mechanical testing impossible at this stage. It is interesting to note, though, that the rubbery film obtained between the two Kapton sheets exhibited very pronounced flexibility. After repeatedly bending the film into a U-shape and releasing the hold, the film repeatedly jumped back to its original flat shape. 

The observed very strong adherence of the hyper-branched polymer to significantly different types of surfaces may be explained by the very high polarity of the tertiary oligo-uret networks, combined with the well-known surfactant property of the multiple PEG branches.

In addition, by proving the feasibility and mechanism of occurrence of these diisocyanate-derived mono-amine- and mono-hydroxy-mediated synthesis pathways, a completely new family of hyper-branched crosslinked polymers may, hence, be further developed, with a wide variety of potential biomedical and pharmaceutical applications.

As also indicated in the Introduction section, despite the very long-known tendency of diisocyanates to side-react with NH groups of urethanes and ureas to form allophanates and biurets, respectively [1,2,3,26], very numerous polyurethane synthesis-related research publications over many decades attributed the occurrence of two, and even multiple, distinct carbonyl-stretching absorbances in the FTIR spectra of polyurethanes to a combined presence and absence of hydrogen bonding between some portions of the urethane groups—without even considering the very possible occurrence of side reactions and, consequently, the possible formation of chemical structures containing additional carbonyl types, thus exhibiting different adjacent carbonyl-stretching absorbance locations [27,28,29,30,31,32,33,34]. 

As also described here above, four recent research works [35,36,37,38] have conclusively demonstrated the highly preferential occurrence of further consecutive side reactions of allophanates and biurets with additional isocyanate groups, resulting in highly complex hierarchical crosslinking networks of tertiary oligo-uret structures. The characteristic FTIR carbonyl-stretching absorbance of this structure was repeatedly demonstrated to accurately and consistently occur at 1687 cm^−1^ in all these recent research works [35,36,37,38] and further in the present research.

The individual carbonyl-stretching FTIR absorbances of urethane [36], allophanate [35], urea [36,37,38], and biuret [37,38] were determined by using different diisocyanate molar ratios (of between 1:1 and 1:6) [35,36,37,38] or by sterically inhibiting side reactions and/or further tertiary oligo-uret formation [36,37,38] and, also, via the deliberate synthesis of a new polymer inherently exhibiting oligo-uret structures in the polymer repeating unit [38]. It was, thus, also demonstrated that the isolated individual carbonyl-stretching FTIR absorbances of urethane groups (at 1717 cm^−1^), urea groups (at 1621 cm^−1^), and biuret groups (at 1637 cm^−1^) each consisted of a single sharp absorbance peak—all of which were not split [36,37,38]. The carbonyl-stretching FTIR absorbance of the allophanate structures, though, exhibited two distinct carbonyl-stretching absorbance peaks, representing the two types of carbonyls in this structure—i.e., the urethyl carbonyl and the uret carbonyl of the allophanate structure (at 1805 cm^−1^ and at 1770 cm^−1^ respectively) [35].

A shifting effect of hydrogen bonding on the location of the urethane carbonyl-stretching FTIR absorbance is commonly suggested to occur in the range of 1705 cm^−1^–1725 cm^−1^, but as two distinct absorbance peaks—one due to the non-hydrogen-bonded urethane carbonyls and the other due to hydrogen-bonded carbonyls [62]. 

The inevitable question that arises here is why a hydrogen bonding-/non-bonding-related peak-splitting effect is mainly reported in relation to diisocyanate-derived polymers, such as polyurethanes and poly(urethane urea)s, and not in other polymers which also exhibit a very high ability of hydrogen bonding, such as polyamides—in which there are no side reactions that can alter the final polymer chemistry. 

In a very recent publication entitled *Infrared Spectroscopy of Polymers XIII: Polyurethanes* [62], the author states the following: “The spectra of polyurethanes are similar to polyamides since both polymer types contain C=O and N-H bonds. Therefore, we will begin with a review of the spectra of polyamides. The structure and infrared spectra of polyurethanes and polymeric amides or polyamides are similar, hence a review of polyamides is in order” [62]. 

Accordingly, in order to further examine the often suggested paradigm of combined hydrogen bonding and non-bonding as being the source of two or multiple distinct FTIR carbonyl-stretching absorbances in polyurethanes, an additional synthesis was hereby performed, leading to NH- and C=O-containing structures, but without the possibility of occurrence of any side reactions. Accordingly, nylon 6,6 polyamide was hereby synthesized via interphase polymerization at room temperature (with adipoyl chloride and hexamethylenediamine).

Absolutely no side reactions occur via the interphase polymerization of this polymer—hence, only one type of carbonyl exclusively exists in the polymer.

Also, highly abundant hydrogen bonding is likely to occur in this polymer, due to the very high content of amide groups along the polymer chain. Nevertheless, as is well-known in all linear (non-crosslinked) polymers, at least a certain degree of chain entanglements is almost inevitably expected to occur. This, along with chain-folding regions, is among the main reasons that crystallizable polymers (nylon 6,6 included) are always semicrystalline—and can never reach full crystallinity (except in some polymeric fibers) [31,32,33,34,35,36,37]. Thus, and for the same reasons, it is highly reasonable and expected that not all the amide groups of the presently synthesized nylon 6,6 would be able to create hydrogen bonds, considering the very short distance range of effective hydrogen bonding (which is only slightly longer than a covalent bond). Accordingly, if the hydrogen bonding/non-bonding carbonyl-stretching FTIR peak-splitting paradigm is valid, as often suggested for polyurethanes, the FTIR spectrum of the nylon 6,6 should exhibit at least two distinct adjacent carbonyl-stretching absorbances—although only one type of carbonyl exists in the polymer.

**Figure 14:**
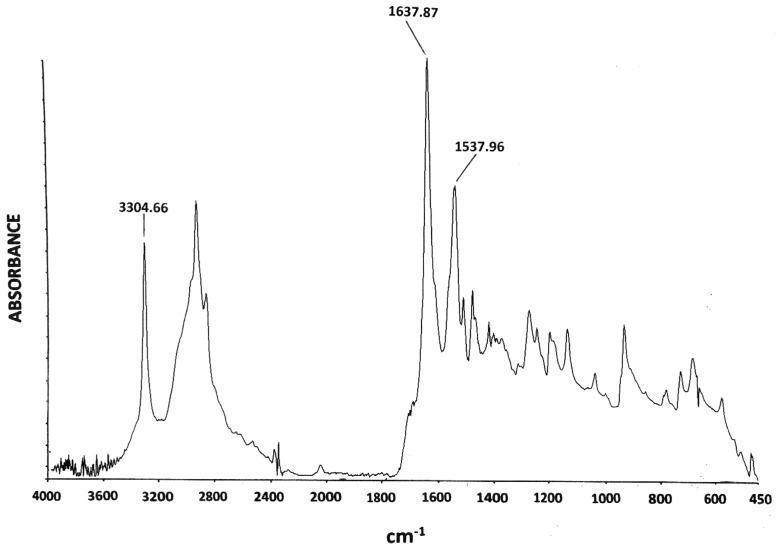
FTIR spectrum of the nylon 6,6 hereby synthesized via interphase polymerization.

Figure 14 exhibits the FTIR spectrum of the nylon 6,6 hereby synthesized via interphase polymerization. A single extremely thin and sharp carbonyl-stretching absorbance appears in the spectrum at 1637 cm^−1^—which strongly confirms that only a single type of carbonyl is present in the polymer, i.e., the amide carbonyl. Also, a single extremely thin and sharp NH-stretching absorbance at 3304 cm^−1^ and a very thin and sharp CNH deformation absorbance at 1537 cm^−1^ are clearly observed in the spectrum of the polymer—again indicating that only one type of NH is present in the polymer, i.e., the amide NH. This strongly confirms that when only one type of carbonyl is present in the polymer—in this case, the amide carbonyl—a single carbonyl-stretching absorbance peak is present in the spectrum.

Hydrogen bonding between amide groups—which is most probably present in nylon 6,6—most probably does cause a shift in the carbonyl-stretching absorbance, but it certainly does not cause any splitting of the peak. As described above, this was also recently demonstrated for diisocyanate-derived polymers containing only urethane [36], only biuret [37], or only urea [37,38] groups, each exhibiting a single sharp carbonyl-stretching FTIR absorbance.

As also observed in the present research, the hydrogen bonding of the non-side-reacted portion of urethane groups did cause a shift in the urethane carbonyl-stretching absorbance (from the usual 1717 cm^−1^ location to 1710 cm^−1^, also exhibiting a thickening or slight shoulder of the peak at around 1717 cm^−1^)—but not the splitting of the peak. The tertiary oligo-uret carbonyl-stretching absorbance at 1687 cm^−1^ did not shift (as it never does) since the structure does not have a polar hydrogen, in view of the fact that all nitrogens in the structure are tertiary nitrogens and, therefore, do not create hydrogen bonds.

**Figure 15:**
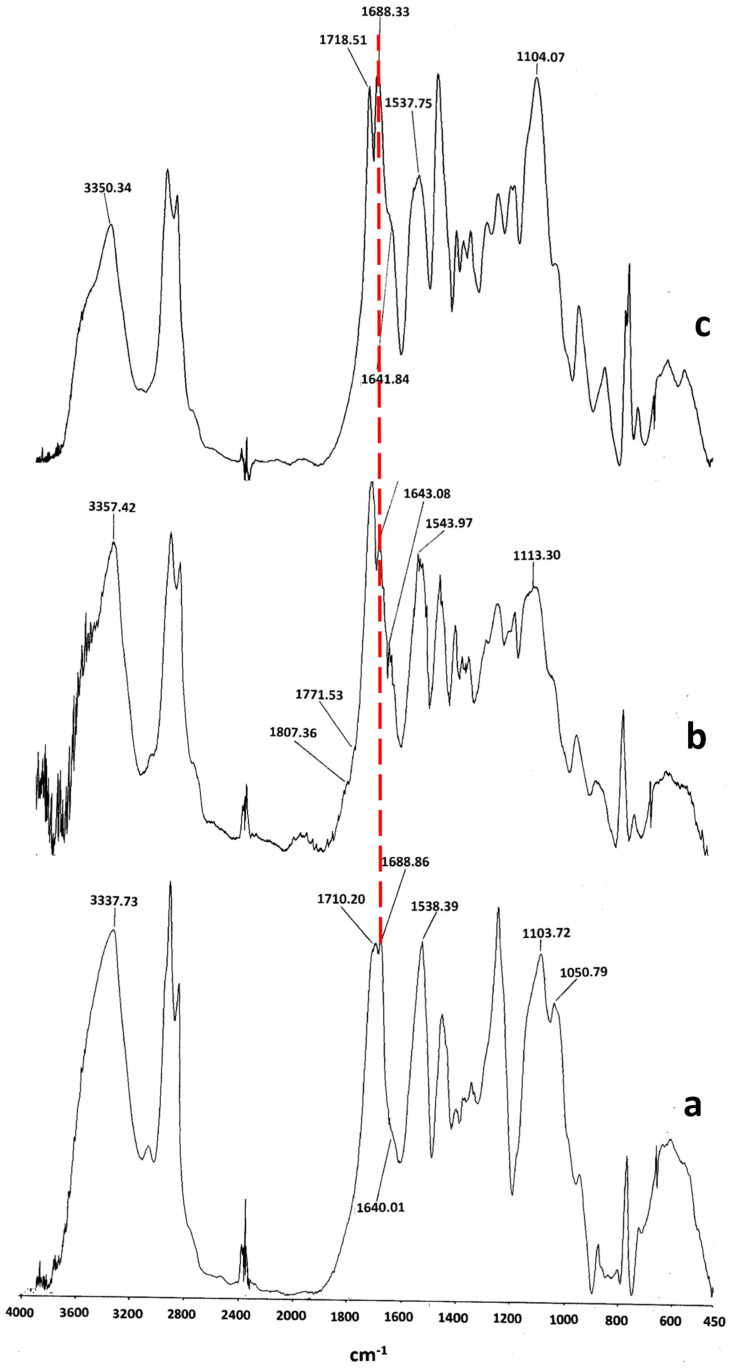
FTIR spectra of the poly(ether urethane) synthesized with HDI and PEG200 at a molar ratio of 1:1 (**a**); the poly(ether urethane) synthesized with HDI and PEG400 at a molar ratio of 1:1 (**b**); the hyper-branched polymer synthesized with HDI and PEG350M at a molar ratio of 1:1 and a –N=C=O:OH molar ratio of 2:1 (**c**). The red dashed line indicates the location of the tertiary oligo-uret carbonyl-stretching absorbance at 1688 cm^−1^, which accurately remains in the same location throughout all the polyurethanes’ spectra (**a**–**c**).

Figure 15 exhibits a summary comparison of the FTIR spectra of the hereby synthesized poly(ether urethane) types, namely the following: the poly(ether urethane), synthesized with HDI and PEG200 at a molar ratio of 1:1 (a); the poly(ether urethane), synthesized with HDI and PEG400 at a molar ratio of 1:1 (b); the hyper-branched polymer, synthesized with HDI and PEG350M at a molar ratio of 1:1—thus exhibiting a –N=C=O:OH molar ratio of 2:1 (c). The tertiary oligo-uret carbonyl-stretching absorbances accurately and consistently appear at 1688 cm^−1^ in all the spectra and are perfectly aligned with the connecting red dashed line, annotated in Figure 15—these resonances are strongly consistent, with the exact same location of the tertiary oligo-uret FTIR carbonyl-stretching absorbance previously reported in polyurethanes, polyureas, hyper-branched polyurea [35,36,37,38], and in poly(hexamethylene oligo-uret) [38]. When viewing the adjacent urethane carbonyl-stretching absorbance, a shift from 1717 cm^−1^ (Figure 15b,c) to around 1710 cm^−1^ (with a slight shoulder/thickening at around 1717 cm^−1^) is observed, which most probably occurs due to hydrogen bonding, which is consistent with and within the very recently reported range for the hydrogen bonding-related shift of the urethane carbonyl-stretching absorbance [60]. The urethane carbonyl-stretching absorbance in the spectrum is observed to shift within this range (Figure 15a)—but no splitting is observed (Figure 15a–c). The strong and sharp absorbance at 1050 cm^−1^ to the right of the main ether absorbance is easily observed in the FTIR spectrum of the poly(ether urethane), synthesized with HDI and PEG200 at a molar ratio of 1:1 (Figure 15a), characteristic of PEG in the crystalline state, as also discussed above. Also, the spectrum of the same polymer exhibits a very much increased intensity of the asymmetric CH-stretching absorbance at 2950 cm^−1^, as compared to the symmetric CH-stretching absorbance at 2850 cm^−1^, which is highly consistent with the very significant chain alignment in this polymer and the consequent parallel extended-chain interaction, predominantly enabling the asymmetric CH-stretching vibration, as also discussed here above. 

The unique structure and properties combination of the presently developed segmented polyurethane, alongside the significantly advantageous single-step synthesis pathway hereby performed, may lead to the further development of segmented polyurethanes of enhanced performance and to improved industrial processing efficacy for a variety of possible applications, ranging from various medical devices to ballistic impact-resistant devices and composite matrices. The mechanisms of the side reactions that occur are demonstrated in the present research, and their effect on the properties of segmented polyurethanes can facilitate the optimization of industrial processes and potentially open a new door to the development of segmented polyurethanes exhibiting a wide range of novel properties and applications. 

## 3. Experimental Methods

### 3.1. Materials

Hexamethylene diisocyanate (purum, ≥98.0%; Sigma) (Sigma-Aldrich Israel Ltd., an affiliate of Merck KGaA, Darmstadt, Germany); PEG MW = 200 (PEG200) (Sigma); PEG MW = 400 (PEG400) (Sigma); Methoxypolyethylene glycol (PEG monomethyl ether) MW = 350 (PEG350M) (Sigma); Ethyl stannous hexanoate (Tin (II) 2-ethylhexanoate 92.5–100%) (Sigma); Adipoyl chloride (98%; Sigma); Hexamethylenediamine (98%; Sigma); Cyclohexane (99.5%; Sigma); KBr powder for FTIR analysis (FTIR grade, ≥99%; Sigma).

### 3.2. Instrumentation

FTIR analyses were performed on a Perkin Elmer Spectrum BX FTIR Spectrometer—with a 4 cm^−1^ instrument resolution. Polymer samples with weights ranging between 8 and 10 mg were shredded with a metal file into small particles and then ground in a clean agate mortar with 140 mg of dried KBr powder until a homogeneous powder was obtained, after which KBr pellets were prepared. FTIR measurements were performed in the 450–4000 cm^−1^ range, in Absorbance mode, and 16 scans were performed during each measurement. 

Solid-state ^13^C NMR spectroscopy measurements were carried out on a narrow-bore Bruker Avance III 400 MHz spectrometer, equipped with a 4 mm BB/1H/19F magic angle spinning (SB MAS) probe. ^13^C CPMAS (cross-polarization magic angle spinning) measurements were performed at a spinning rate of 8 KHz with a 2.9 μs ^1^H 90° pulse, 2 K data points, and with a 2 ms ramped-CP period. During both the acquisition and the 3 s cycle delay between acquisitions, proton decoupling using the SPINAL (small phase incremental alteration) composite pulse sequence in a field of 86 KHz was conducted. Approximately 200 mg of polymer per measurement were used.

Differential scanning calorimetry (DSC) analyses were performed on a Mettler STARe SYSTEM DSC3 (Switzerland), equipped with a DSC sensor FRS 5 and an Intracooler Isolation Kit. Small polymer samples were cut and accurately weighed (in the range of 5–15 mg) in an aluminum crucible and then sealed with a preliminarily perforated aluminum lid and placed on the sample site. An identical sealed aluminum crucible was placed on the reference site. The measurements were performed in the range of 0–200 °C at a heating rate of 10 °C/min and under nitrogen gas flow.

Tensile mechanical tests were performed on an AGS-X+250 Shimadzu (Kawasaki, Japan) universal testing machine, equipped with a 10 KN load cell (C1 500 AGS-X) at a working range of 20–10,000 N and an accuracy of ±1% and 5 KN screw-type grips (with embedded file teeth-type grip face). Seven samples of the poly(ether urethane) elastomer were measured. The samples size was approximately 150 mm in length, 10 mm in width, and 1.5 mm in thickness. The exact widths and thicknesses of the samples were measured using a digital caliper and a micrometer, respectively, and fed into the instrument software. Measurements were performed at a 5 mm/min speed.

Optical microscopy observations of the polymers were carried out using a Nikon H550S optical microscope (Nikon, Tokyo, Japan). The microscope is equipped with an INVENIO 5SCIII 5M Pixel USB3 color digital camera and connected to Deltapix Insight 2.5 software. During microscopy observations, only the focus and light intensity were adjusted. 

### 3.3. Polymer Syntheses

Polyurethane syntheses were performed in a 150 mL glass reactor under motor-driven mechanical stirring with a Teflon-coated stirring rod and under a continuous nitrogen flow atmosphere. Synthesis temperature was maintained at approximately 80 °C using an electrically heated oil bath with an immersed thermometer. All synthesis equipment in contact with the reactants (i.e., glass reactor an stirring rod) was preliminarily dried in an oven at 105 °C; polyethylene glycol was dried at 105 °C under vacuum and magnetic stirring for 90 min, prior to synthesis; the HDI was removed from refrigeration prior to synthesis, for a period long enough to reach room temperature, in order to prevent atmospheric water vapor condensation when opening the container—in order to maintain dry reaction conditions. Syntheses with PEG200 or PEG400 soft segments were performed in bulk at a PEG–HDI 1:1 molar ratio, and calculations were performed to obtain a final weight of 50 g of polymer. PEG was weighed directly into the reactor in view of its significant viscosity. HDI was weighed separately and added to the reactor under mild mechanical stirring, after which 0.3 mL of ethyl stannous hexanoate catalyst was added to the mixture and stirring speed was increased to the maximum speed that did not cause the splattering of the reactor content—and constantly maintained until all reactor content turned into a rubbery solid polymer. At this stage, the very quick removal of the still-hot rubbery polymer was required, since upon cooling, the significantly crosslinked polymer exhibits high strength and an extremely strong adherence to the reactor glass wall and, thus, the removal of the polymer from the reactor becomes practically impossible—if not instantly removed while still hot. 

The synthesis temperature was chosen following several optimization synthesis trials, indicating that performing the synthesis at lower temperatures resulted in significant premature polymer solidification inside the reactor, which led to the premature inefficiency of the mixing process before reaction completion and also led to the extreme difficulty of removing the polymer from the reactor. Performing the synthesis in an oil bath at a temperature of 80 °C kept the solidifying reactor content at a relatively manageable viscosity to enable continued mixing and the eventual removal of the polymer from the reactor (though still with significant effort).

Polyurethane synthesis requires the presence of a catalyst (as opposed to polyurea synthesis with a diamine, which does not necessarily require the presence of a catalyst). Nevertheless, almost the minimum concentration of catalyst, which is still effective during bulk synthesis, was hereby chosen, since significant amounts of residual catalyst in biomedical poly(ether urethanes) are known to catalyze the in vivo oxidative degradation of the polyether soft segments of these polymers.

During the hot polymer removal process from the reactor, several thin and flat strips were manually shaped by shape-fitting the still-hot polymer against a pre-formed metal wire template of measured length and width (of 150 mm in length and 10 mm in width) for subsequent mechanical tests. Since the polymer is significantly crosslinked, reshaping the polymer after cooling is not possible under any conditions. 

The synthesis of hyper-branched polymers with PEG monomethyl ether 350 (PEG350M), was performed in bulk at a PEG350M–HDI molar ratio of 1:1 (which actually constitutes a N=C=O:OH molar ratio of 2:1) under the exact same conditions and synthesis process as described above. Syntheses were calculated to obtain a final weight of 50 g of polymer. The synthesis resulted in a relatively high-viscosity amorphous polymer, the physical properties of which gradually increased at room temperature within 3–4 months following synthesis completion.

All synthesized polymers were stored at room temperature for at least three months after synthesis completion before the characterization of the polymers, since it was previously demonstrated that polyurethanes assume their final properties approximately three months following synthesis completion [36]. The long-term periodic FTIR analysis of polyurethanes containing tertiary oligo-uret structures during a three-month-long incubation period at room temperature clearly showed a consistent increase in the tertiary oligo-uret carbonyl-stretching intensity at 1687 cm^−1^ and a concomitant consistent decrease in the free isocyanate group absorbance intensity at 2270 cm^−1^ as a function of time—with a total disappearance of the isocyanate absorbance and concomitant maximum intensity of the tertiary oligo-uret carbonyl absorbance reached after approximately three months of incubation [36]. During this incubation period, the physical properties of the polymer also gradually and significantly increased. 

The synthesis of nylon 6,6 was performed via interphase polymerization at room temperature. The aqueous phase consisted of 9.5 mL of distilled water, 0.5 g of hexamethylenediamine (HMDA), and 10 drops of NaOH 5N (the NaOH was added to neutralize the HCl that arises from the condensation reaction during synthesis). The organic phase consisted of 9.5 mL of cyclohexane and 0.5 mL of adipoyl chloride. Each of the phases was prepared in a separate glass beaker. The organic phase was very slowly poured over the aqueous phase in order to prevent agitation. At the instant of phase contact, a polyamide (nylon 6,6) membrane was instantly formed at the interface between the two phases. The membrane was continuously manually pulled out of the system using tweezers. The nylon 6,6 membrane was manually straightened and placed over the rim of a clean glass beaker and then dried at 40 °C under vacuum for approximately 12 h. A very thin film of nylon 6,6 was thereby obtained, which was used for FTIR analysis.

## 4. Conclusions

Polyurethane elastomers are typically achieved through a two-step synthesis finally resulting in segmented block copolymers comprising very long soft segments that provide elasticity and significantly long hard segments that provide strength.

The present research focused on the design of a highly efficient single-step synthesis of a new segmented polyurethane consisting of very short soft and hard segments, crosslinked by preferentially side-reacted hierarchical tertiary oligo-uret network structures, thus exhibiting significant strength, elasticity, and toughness.

A segmented poly(ether urethane) was synthesized by reacting PEG200 with HDI at a 1:1 molar ratio, the theoretically linear structure of which consisted of a very short soft segment of only four ethylene oxide units and a hard segment of only two urethane groups.

Despite the theoretically linear structure of the newly synthesized polymer, both FTIR and solid-state ^13^C NMR spectroscopy analyses indicated a highly non-linear structure, exhibiting a quasi-equal presence of urethane groups and tertiary oligo-uret network structures in the resulting polymer. This is highly consistent with a mechanism of the preferential consecutive side-reaction of isocyanate groups with secondary nitrogens in a hierarchical consecutive pattern, rather than creating new allophanate structures with the remaining non-side-reacted urethane groups in the polymer and even rather than creating new urethane groups. 

Although PEG200 itself is an amorphous liquid which cannot crystallize under any conditions, the DSC analysis of the resulting polyurethane exhibited a significant endotherm at a temperature characteristic of the melting of the crystallized PEG. This was further also confirmed in the FTIR spectrum of the polymer by the strong presence of an additional C–O–C stretching absorbance in a well-known location that is characteristic of crystalline PEG. Thus, the data obtained suggest the possible presence of crystalline domains mainly consisting of PEG chains. It was hereby deduced that the mechanism of the PEG200 soft segment crystallization in this polymer was the parallel extended-chain mechanism, through the orientation of the PEG segments anchored in place by the crosslinking structures at their extremities—and not via a chain-folding crystallization mechanism. The suggested parallel extended-chain mechanism was also highly consistent with, and confirmed by, the tensile stress–strain curve profile and the relatively low elongation at break (as compared to average elastomers), which was highly consistent with the state of the extended-chain pre-alignment of the polymer soft segments. The optical microscopy photographs of the polymer indicated very significant chain orientation, also exhibiting occasional regions of fibrillation.

Tensile mechanical testing exhibited a combination of significant strength, elasticity, and toughness. In view of the very short soft and hard segments of the present polymer, it is hereby deduced that the flexibility of the complex hierarchical tertiary oligo-uret crosslinking network structures strongly contributed to both the elasticity and strength of the resulting segmented polyurethane, which is in significant agreement with the purpose and objectives of the present research.

Increasing (doubling) the length of the polymer soft segment at constant hard segment length led to a decreased efficacy of tertiary oligo-uret formation—and mainly a decreased efficacy of the secondary crosslinking of the formed tertiary oligo-uret structures, due to the increased distance between the crosslinking sites—which resulted in very poor properties of the resulting polymer.

The preferential occurrence mechanism of the above-described side reactions and of the resulting hierarchical tertiary oligo-uret networks formation were hereby further confirmed and elaborated through the design and synthesis of a completely new type of hyper-branched polymer, by reacting the same diisocyanate (HDI) with a mono-hydroxy-terminated PEG, instead of the diol-terminated PEG used in the synthesis described above. Both FTIR and solid-state ^13^C NMR spectroscopy analyses indicated a very strong presence of tertiary oligo-uret structures, along with a very significant amount of remaining non-side-reacted urethane groups. This unambiguously indicated that the N=C=O groups in the system preferentially further consecutively side-reacted with the significantly smaller amount of already formed allophanate groups, instead of forming additional new allophanates with the remaining non-side-reacted urethane groups.

In order to further demonstrate that the occurrence of two or more distinct carbonyl-stretching FTIR absorbances are most probably due to the occurrence of side reactions and not due to a combined presence of hydrogen bonding and non-bonding, as commonly suggested for polyurethanes, an additional synthesis was hereby performed, leading to NH- and C=O-containing structures, with abundant hydrogen bond formation ability but without the possibility of the occurrence of any side reactions. Accordingly, nylon 6,6 polyamide was hereby synthesized via interphase polymerization at room temperature. A single extremely thin and sharp carbonyl-stretching absorbance was obtained in the spectrum, strongly indicating that the combined presence of hydrogen-bonded and non-bonded structures (which are most probably present in this polymer, as also in polyurethanes) may of course cause a shift of the absorbance peak but most probably does not cause any peak-splitting of the FTIR carbonyl-stretching absorbance, as was previously often attributed to polyurethanes.

The structure–property relations and reaction mechanisms demonstrated in the present research can facilitate the design of new polyurethanes of enhanced performance and processing efficacy for a variety of novel applications, ranging from various medical devices to ballistic impact-resistant devices and as composite matrices. 

The novelty of the present research pertains to advantageously and purposefully using the recently discovered tertiary oligo-uret-forming side reactions in the design of new non-linear polyurethane elastomers, in order to obtain the desired properties of polyurethane elastomers, through the following: (1) shorter single-step syntheses; (2) using very short hard segments in combination with tertiary oligo-uret networks and consequently using much smaller amounts of diisocyanate; and (3) using non-aromatic diisocyanates, which will be highly beneficial in terms of both safety and environmental considerations and also significantly reduce industrial manufacturing costs. The present research also demonstrated that the optimal efficacy of tertiary oligo-uret development and its efficient secondary crosslinking occurs when using very short soft segments; this efficacy decreases with increasing soft segment length. Thus, the importance and efficacy of using very short soft and hard segments in these polymers was hereby demonstrated. 

A completely new type of hyper-branched polymer was hereby synthesized for the first time by advantageously using the tertiary oligo-uret-forming side reactions, which were also used to conclusively demonstrate the occurrence of these side reactions, without which this synthesis would not have been possible. 

In addition, the present research presents a new approach in the diagnostic interpretation of the FTIR carbonyl-stretching absorbances in polyurethanes, as related to the occurrence of side reactions.

## Figures and Tables

**Table 1:** Tensile mechanical tests performed on the poly(ether urethane), synthesized with HDI and PEG200 at a molar ratio of 1:1.

Sample No.	Ultimate Tensile Strength (MPa)	Young Modulus (MPa)	Strain at Break (%)
1	26.4	104.2	162.7
2	32.3	125.1	177.4
3	26.9	117.0	183.8
4	27.5	58.2	223.3
5	33.5	124.9	172.2
6	32.2	139.0	177.7
7	22.9	102.9	184.2
Average/Std.	28.8/3.6	110.2/24.2	183.0/17.8

## Data Availability

Data are contained within the article.
